# Concept2Brain: an AI model for predicting neurophysiological responses to text and pictures

**DOI:** 10.1038/s41467-026-75653-x

**Published:** 2026-07-23

**Authors:** Alejandro Santos-Mayo, Faith Gilbert, Arash Mirifar, Anna-Lena Tebbe, Ruogu Fang, Mingzhou Ding, Andreas Keil

**Affiliations:** 1 Laboratory of Brain, Body, and Behavior, University of Florida, Gainesville, FL USA; 2 Department of Psychology, University of Florida, Gainesville, FL USA; 3 Center for Cognitive and Computational Neuroscience, Complutense University of Madrid, Madrid, Spain; 4 J. Crayton Pruitt Family Department of Biomedical Engineering, University of Florida, Gainesville, FL USA

**Keywords:** Perception, Neural encoding

## Abstract

Evolving methods rooted in artificial intelligence (AI) offer new opportunities for linking human behavior and experience to brain function. Here, we introduce the Concept2Brain model, a deep network designed to generate synthetic electrophysiological responses to semantic/emotional information conveyed through pictures or text. Leveraging AI solutions like CLIP from OpenAI, the model generates a representation of a stimulus and maps it into an electrophysiological latent space. We demonstrate that this openly available resource generates synthetic neural responses that closely resemble those observed empirically. The Concept2Brain model is provided as a web service tool for creating open and reproducible EEG datasets by predicting brain responses to any semantic concept or picture. Beyond its practical applications, it also paves the way for AI-driven brain activity modeling, offering new possibilities for studying how the brain represents the world.

## Abstract (web summary)

Advancing toward human brain simulation remains a major challenge. Here, the authors introduce Concept2Brain, an AI web tool that predicts neural activity, thus generating synthetic and reproducible EEG datasets from text or picture inputs.

## Introduction

One of the most significant scientific milestones of the 1920s was the recording of the first human scalp electroencephalogram (EEG) in 1924 by Hans Berger, which laid the foundation for understanding human brain processes^1^. EEG has since evolved into a versatile research tool with applications extending beyond neuroscience into fields such as economics, marketing^2^, rehabilitation^3^, and human-computer interaction^4^, including brain-computer interface (BCI) technology^5^. Recently, artificial intelligence (AI)-driven approaches for analyzing EEG data have emerged^6–8^. These methods link EEG signals to behavior, enhancing real-time brain decoding^6,9–11^, and optimizing BCI performance^12,13^. Synergy between EEG and AI reflects a broader trend, showing an exponential rise in AI-driven solutions, applied to both scientific research and real-world problems.

While earlier AI approaches in neuroscience focused on analyzing brain activity for data classification and computational modeling, more recent work involves creating artificial systems that generate or reproduce brain-like data in response to a given stimulus^12–14^. Instead of decoding brain activity to classify or reconstruct a given stimulus, event, or condition, these approaches encode external stimuli to predict the corresponding brain activity. Well-validated encoding models provide a foundation for testing hypotheses in simulated environments, thereby advancing our understanding of neural processes. However, despite their potential, encoding models for EEG remain relatively underexplored.

To address this problem, the present report introduces Concept2Brain, a deep neural network architecture designed to generate synthetic EEG signals from either pictures or text. Specifically, the model has been trained to reconstruct EEG signals associated with the perception of semantically rich scenes varying in content. EEG signals that are time-locked to specific sensory or behavioral events can be measured through event-related potentials (ERPs). ERPs are obtained by repeating those events many times and averaging the event-locked EEG voltage time series in the time domain^15–17^. Beyond ERPs, EEG signals also contain oscillatory signals, many of which are seen across animal and human studies^18^. Specific aspects of the electrophysiological signals discussed above have been reliably linked to the processing of semantic concepts, including those varying in object category or emotional content^19–22^. If information is systematically shared between electrophysiological responses (electrophysiological latent space; Supplementary Fig. 1.1) and real world concepts as expressed in media and text (conceptual latent space in Supplementary Fig. 1.2), then it should be possible to translate semantic content from the domain of graphical or language representations into the domain of brain activity (see Supplementary Fig. 1.3).

The Concept2Brain model is designed to translate stimulus content into brain activity, using real-world conceptual information to generate synthetic EEG data. The synthetic data are a model-based prediction of the electrophysiological response of an observer’s brain to a specific stimulus and thus resemble ERPs. Concept2Brain is available as an open cloud platform (http://concept2brain.brainbodybehavior.science). Brain responses for different synthetic participants can be generated based on user-entered prompts, which can be written text or digital pictures (Supplementary Fig. 1.4). By generating large, diverse, and customizable datasets, this technology addresses temporal and financial barriers associated with costly and difficult EEG/ERP data acquisition. Thus, it enables researchers from a wide spectrum of backgrounds and interests to generate simulated data, which facilitates the initial exploration of novel hypotheses, simulation studies, as well as power analyses. The present report details the generation and validation of the Concept2Brain model and discusses its potential applications.

## Results

### Model training and cross-validation

The Concept2Brain model consists of two deep neural networks, trained on empirical data in response to naturalistic picture sets, and validated through a comparison between the empirical and synthetic data.

**Fig. 1:**
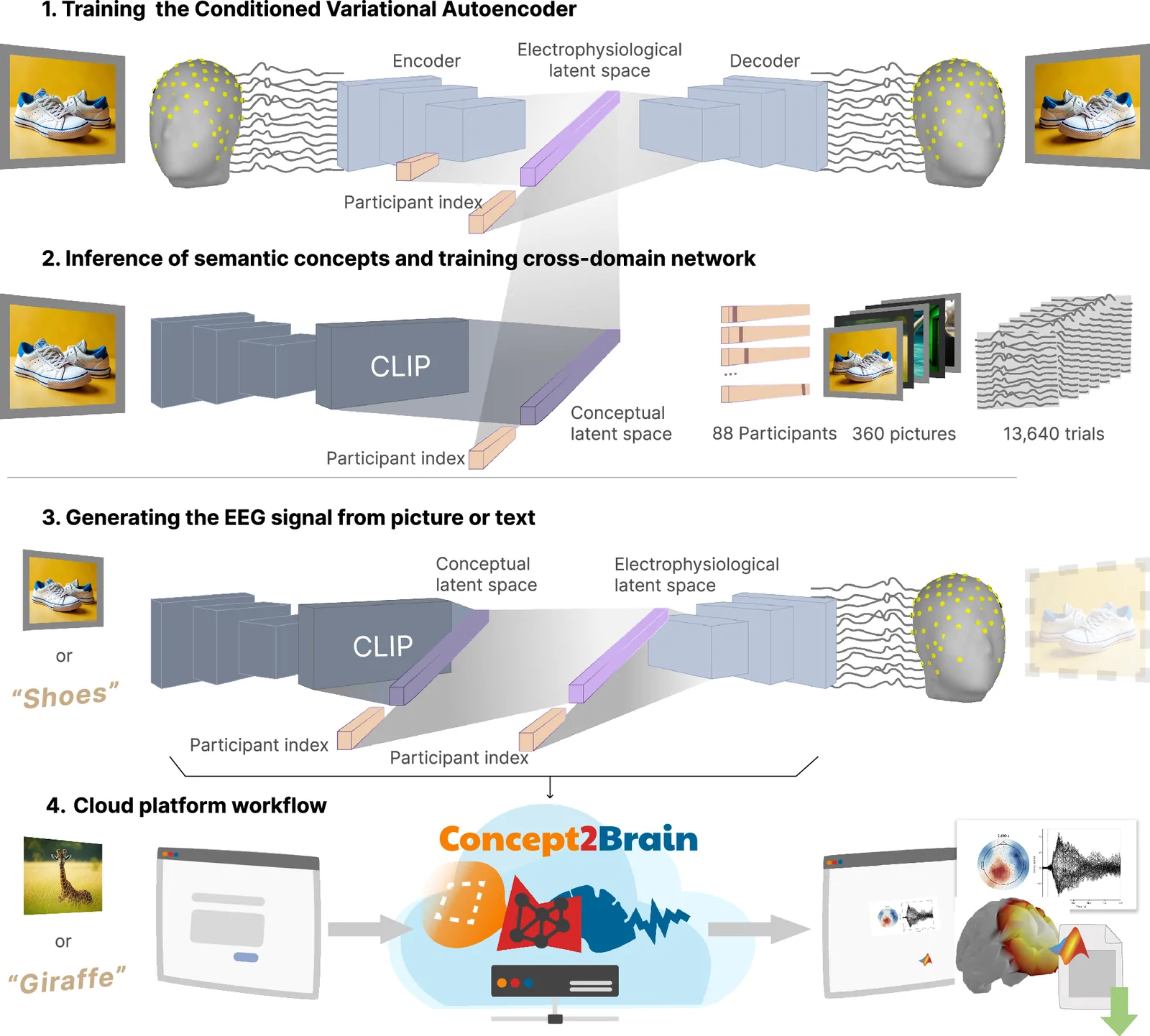
Development and deployment scheme of the Concept2Brain model. 1) Training phase of the cVAE architecture for extracting the electrophysiological latent space from the EEG datasets. The blue boxes, light purple vector and light orange vectors refer to the convolutional neural layers, the bottleneck of the network and the vector of participant ID, respectively. 2) The CLIP model by OpenAI is used to parse the semantic content of pictures into a multidimensional latent space. Then, a neural network is trained as a cross-domain model, linking the conceptual and electrophysiological latent spaces. 3) The Concept2Brain model leverages cross-domain cVAE models to translate pictures or text into synthetic EEG signals. 4) Concept2Brain is deployed in a cloud-computing platform for open-access use of the model without any hardware nor software requirements.

To create the model, an initial neural network was produced using a conditional variational autoencoder (cVAE, Supplementary Fig. 1.1), which compresses high‑dimensional EEG signals into a compact latent space that captures the relevant information. Unlike a standard VAE, the cVAE incorporates a conditioning variable, here the participant, into both encoder and decoder networks, thereby enabling the reconstruction of EEG patterns for specific simulated participants (see *Methods*). Once trained, the model can generate new EEG time series based on the information that is entered into this latent space. Importantly, the synthetic time series output by Concept2Brain is regularized through the entire architecture and thus possess higher signal-to-noise ratios than do single trials of empirically recorded EEG. Thus, the output of Concept2Brain resembles trial-averaged empirical signals (i.e., ERPs). The Concept2Brain model also contains a cross-domain network that translates a conceptual latent space into the electrophysiological latent space of the cVAE. In its present state, the architecture uses CLIP, an AI-based tool, for translating pictures or text into the conceptual latent space (Supplementary Fig. 1.2). The trained network allows users to generate a synthetic EEG response to input from pictures or text, for each virtual participant. Because the architecture was trained on picture-evoked signals, the simulated electrophysiological responses to text should be treated with heightened caution. Fig. 1

**Fig. 2:**
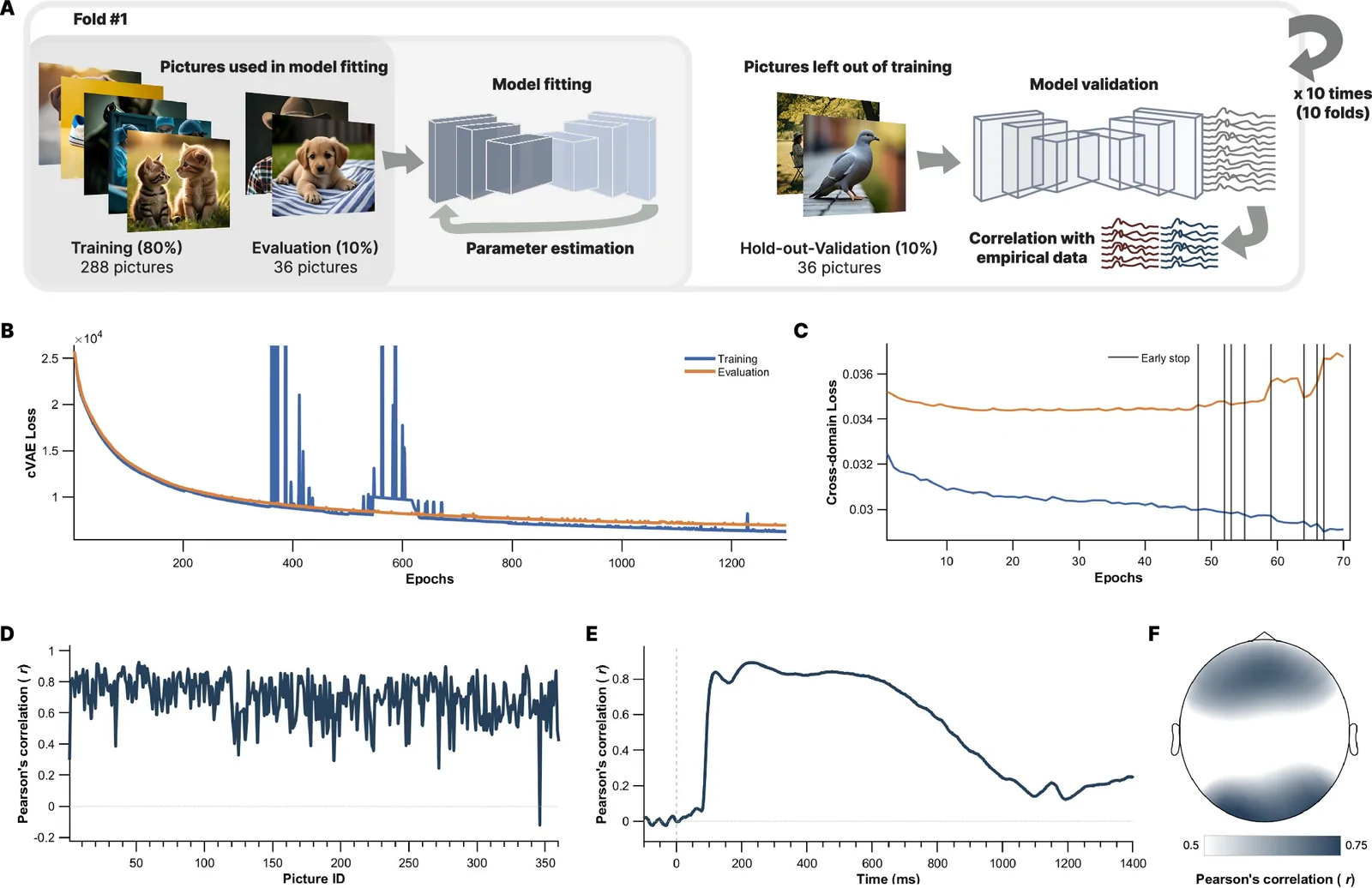
Cross-validation of the Concept2Brain model following a 10-fold cross-validation procedure with held out pictures. **A** Shows how the cross-validation was performed to test the generalization capability of the AI architecture. First, 288 pictures (80% of total) were used as a training dataset to estimate the parameters of both the cVAE and cross-domain models. In parallel, 36 pictures (10%) served as evaluation set to quantify the model’s performance. Once the model was fitted, parameters were frozen and 36 pictures (10%) held-out during the previous phase were fed into the model to measure its generalization capability, by correlating the synthetic and empirical participants. This procedure was repeated 10 times (folds) varying the pictures in each set to cross-validate the model with every picture. **B** The plot shows the median of the loss functions of the training (blue) and evaluation (orange) datasets across 1300 epochs during the fit of the cVAE model. In this phase, a few epochs showed gradient explosions, a common instability of the network during parameter estimation in deep learning training. **C** The plot shows the median loss functions of the training (blue) and evaluation (orange) datasets during the fit of the cross-domain model. Here, generalization was maximized by setting up an early stop (black vertical lines), which stops the training model when the evaluation loss function starts to rise as product of network overfitting. **D** Once the 10 folds were calculated, Pearson’s correlation coefficients were calculated between the synthetic and empirical EEG data for each picture, to quantify how EEG traces to each held-out picture resembled the empirical data. **E** The plot shows the Pearson correlation coefficients across time. Note the higher similarity between modeled and empirical data at latencies consistent with visuocortical and emotional scene responses. **F** The topographical plot shows the Pearson correlation coefficients distributed across the EEG sensor array. Highest correlations were found at occipital electrodes, consistent with the expected impact of posterior visuocortical areas on the topography of the electrophysiological signals.

The Concept2Brain architecture was trained using two EEG datasets (each including 129 channels) in which 88 observers viewed a total of 360 unique naturalistic photographs depicting a wide spectrum of semantically rich information (see Methods section below). During the training phase, data segments, each containing EEG data when viewing one picture, were input into the Concept2Brain model, along with the CLIP-based dimension-reduced picture data. To measure the generative validity of the model, we conducted a 10-fold cross-validation procedure (see Fig. 2A). In each fold, data from 80% (288) and 10% (36) of the pictures were used to train and evaluate the model. First, the cVAE was trained only with the first 80% of pictures, aiming to ensure validity of the electrophysiological latent space. Figure 2B shows the loss function of the cVAE training across 1300 learning epochs. Next, the cross-domain network was trained using the same cross-validation split. Figure 2C shows the fitting process in which the network learns to predict the electrophysiological latent space based on the CLIP model output. The evaluation set was used to control whether the cross-domain network overfit the data. If overfitting was detected, there was an early stop function implemented in the training phase (represented by black lines in Fig. 2C).

Once the model was fully trained, we tested its validity by computing synthetic data using the validation pictures held out of the prior stage and comparing them with empirical recordings using Pearson correlation coefficients. First, we measured the similarity between the synthetic and empirical EEG signal of the 88 participants for each one of the validation pictures left out of the training phase (Fig. 2D). Pearson correlations demonstrated satisfactory generalization performance, with most correlations exceeding 0.6. Surprisingly, picture number 346 showed a correlation of −0.2. This picture’s erotic content may not have been fully captured by the CLIP model, due to content-safety guardrails, thus distorting its dimensional representation. Next, we analyzed the similarity between empirical and synthetic signals in the time domain for the same held-out pictures (Fig. 2E). Crucially, correspondence between empirical and synthetic signals starts at around 100 ms where visuocortical processes start to affect the EEG signal (e.g., Luck, 2012). Substantial correspondence between empirical and synthetic data continues until the end of the trial, fading out after 700 ms. The observed topographical distribution of correlations is expected for a response to visual stimuli, including the projection of occipital dipolar voltage fields to frontal sensors (Fig. 2F).

### Model performance and data reconstruction

**Fig. 3:**
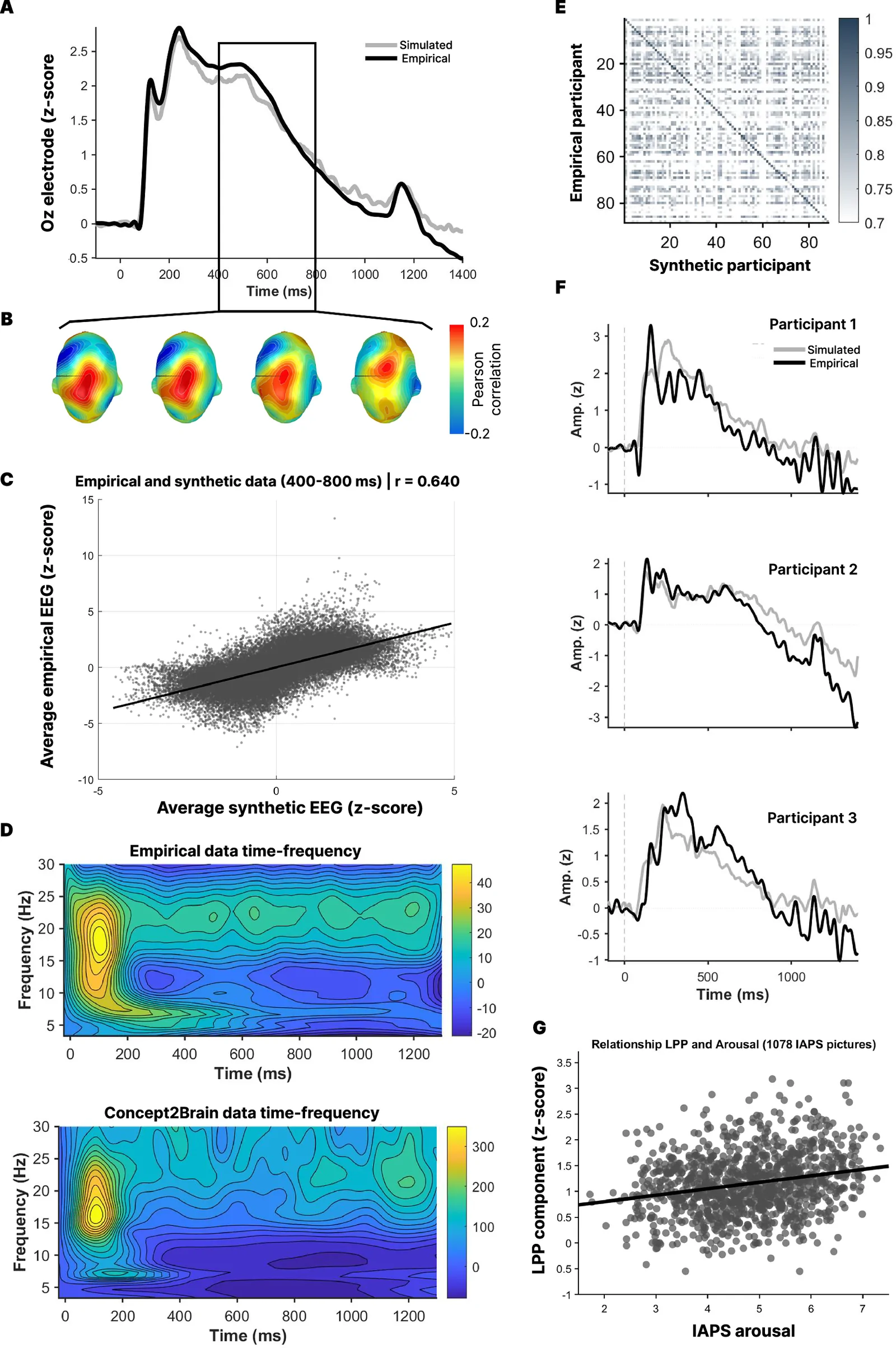
Data reconstruction and participant-level reproducibility of the Concept2Brain model. **A** Shows in black the time series obtained from the empirical EEG signal (average of both datasets), and in gray the time series generated by the Concept2Brain model with the same pictures, both obtained from mid-occipital electrode site Oz. **B** Topographical distribution of Pearson’s correlation during the time range between 400 ms and 800 ms, which in picture viewing studies is associated with the so-called late positive potential (LPP) component. **C**: Scatter plot showing the correlation between empirical and synthetic signals. Each black dot represents an individual time and electrode value for each of the 88 participants. **D** Time-frequency representation of the baseline-adjusted changes in EEG spectral composition upon picture presentation, for empirical (top) and synthetic (bottom) EEG signals. Characteristic oscillatory components such as the initial rise and subsequent reduction of amplitude in the alpha frequency range (around 10 Hz) are visible in both signals. **E** Pairwise correlation matrix between averaged EEG activity of empirical participants and synthetic participants. Larger correlations are distributed along the diagonal, supporting the participant-level fidelity of Concept2Brain. **F** EEG timeseries of both the empirical signal (black line) and the Concept2Brain output (gray line) for the 3 first participants from electrode Oz. **G** Pearson’s correlation coefficient between normative emotional arousal ratings from the International Affective Picture System (IAPS), obtained in large standardization samples from the general population, and the synthetic LPP component generated for 1078 IAPS pictures. In terms of effect size and direction, this same relation has been widely reported in research with emotional pictures. The LPP component was scored by averaging the synthetic voltage at electrode Pz across a time segment from 400 to 800 ms post-onset and across the 88 participants.

After this initial validation of the model training and validation, we further evaluated data recovery by measuring the model’s performance when reproducing the EEG signal based on all pictures contained in the original training set, as well as new pictures not used during training. Model performance was evaluated by quantifying the presence of common ERP components, time-frequency patterns, a comparison of individual participant-participant mapping between empirical and synthetic data, and the correspondence between synthetic brain data and empirical behavioral data. Figure 3 shows the direct comparison between empirical and synthetic EEG signal and a model validation using the IAPS dataset.

The data recovery analysis indicated a high correlation between empirical and synthetic data. First, the ERP response derived from the empirical EEG data and the Concept2Brain output show similar time courses and waveforms (Fig. 3A). Common ERP components such as P100^23,24^, N200^17^, P300^25^ and the late positive potential (LPP)^19,26^ are readily observed in the synthetic data, mirroring the waveform morphology of the empirical data. Next, we examined data reproducibility in space and time. To this end, Pearson’s correlations across the 88 participants were computed for each channel and time point. The highest correlations between empirical and synthetic signals were found from 400 to 800 ms post-picture onset (see Fig. 3B). During this time window, the reconstruction accuracy shows activity in parietal sensors. Thus, both the latency and topography of the best-reconstructed synthetic data match the Late Positive Potential (LPP) component of the ERP—an established ERP metric of emotion perception^19,26,27^. Thus, the Concept2Brain model effectively uses the spatio-temporal electrophysiological information to accurately reproduce a signal corresponding to the semantic-emotional meaning of the stimulus for the observer. In addition, we conducted a data recovery analysis, which correlated all estimated synthetic values with the corresponding empirical EEG values across all channels and time points, for each of the 88 participants. This analysis also revealed a strong correspondence between empirical and synthetic data (Pearson’s *r* = 0.640, *p* < 0.001; Fig. 3C).

As a further validation step, we conducted a time-frequency analysis on both empirical and synthetic EEG data, to examine the extent to which Concept2Brain reproduces the time-varying changes in oscillatory responses to naturalistic pictures. Figure 3D shows that major oscillatory patterns are shared between empirical and synthetic data, including the prominent amplitude reduction of alpha-band (10 Hz) activity after 300 ms. To measure the similarity between empirical and synthetic time-frequency properties, Pearson’s correlations were computed across all time and frequency points. Results (Pearson’s *r* = 0.724, *p* < 0.001) indicate a strong similarity between both datasets. Taken together, these analyses demonstrate the capability of the Concept2Brain model to reproduce key features of empirical electrophysiological signals, although the lack of single-trial variability in response to the same stimulus constrains the use of the data for many advanced single-trial analyses.

One of the main strengths of the Concept2Brain model lies in its capability to replicate participant-level signal characteristics. Therefore, we estimated the participant-wise correspondence between each one of the 88 empirical participants and their synthetic counterparts (Fig. 3E). All participant-wise comparisons showed a maximum correlation coefficient on the diagonal, indicating that EEG data from corresponding synthetic and empirical participants converged. Figure 3F shows empirical and generated signals from the first three participants, with strong inter-individual differences in ERP morphology—a well-established observation in neurophysiological studies with humans^28–30^. These differences highlight the importance of the conditioned component of the cVAE, for accurately reproducing participant-level variability.

Next, we generated synthetic EEG activity of 88 participants in response to all standardized emotional (IAPS) pictures that were not used in the training phase of Concept2Brain. As a result, we obtained 1078 electrophysiological responses to a wide range of pictures, taken from the IAPS database. Previous work^20,31^ has demonstrated a medium-sized positive linear relationship between the amplitude of the LPP component evoked by a given picture and self-reported emotional arousal. In line with this prediction, the out-of-sample LPP component predicted by Concept2Brain (Pz electrode, 400 ms to 800 ms), averaged across all synthetic participants, was correlated with the normative arousal rating of the corresponding 1078 pictures. Results showed a significant Pearson correlation of *r* = 0.23 (*p* < 0.0001) between the LPP and the arousal ratings across the 1078 pictures (Fig. 3 G).

### Out of sample validation using external datasets

The main goal of the Concept2Brain model is to offer a tool capable of generating new realistic EEG datasets. Therefore, synthetic datasets generated by the model should present similarities with empirical EEG datasets. To evaluate the model’s validity, we applied Concept2Brain to two publically available data sets providing data and stimuli. Both datasets comprise picture stimuli that were not seen by the present model during the training phase.

**Fig. 4:**
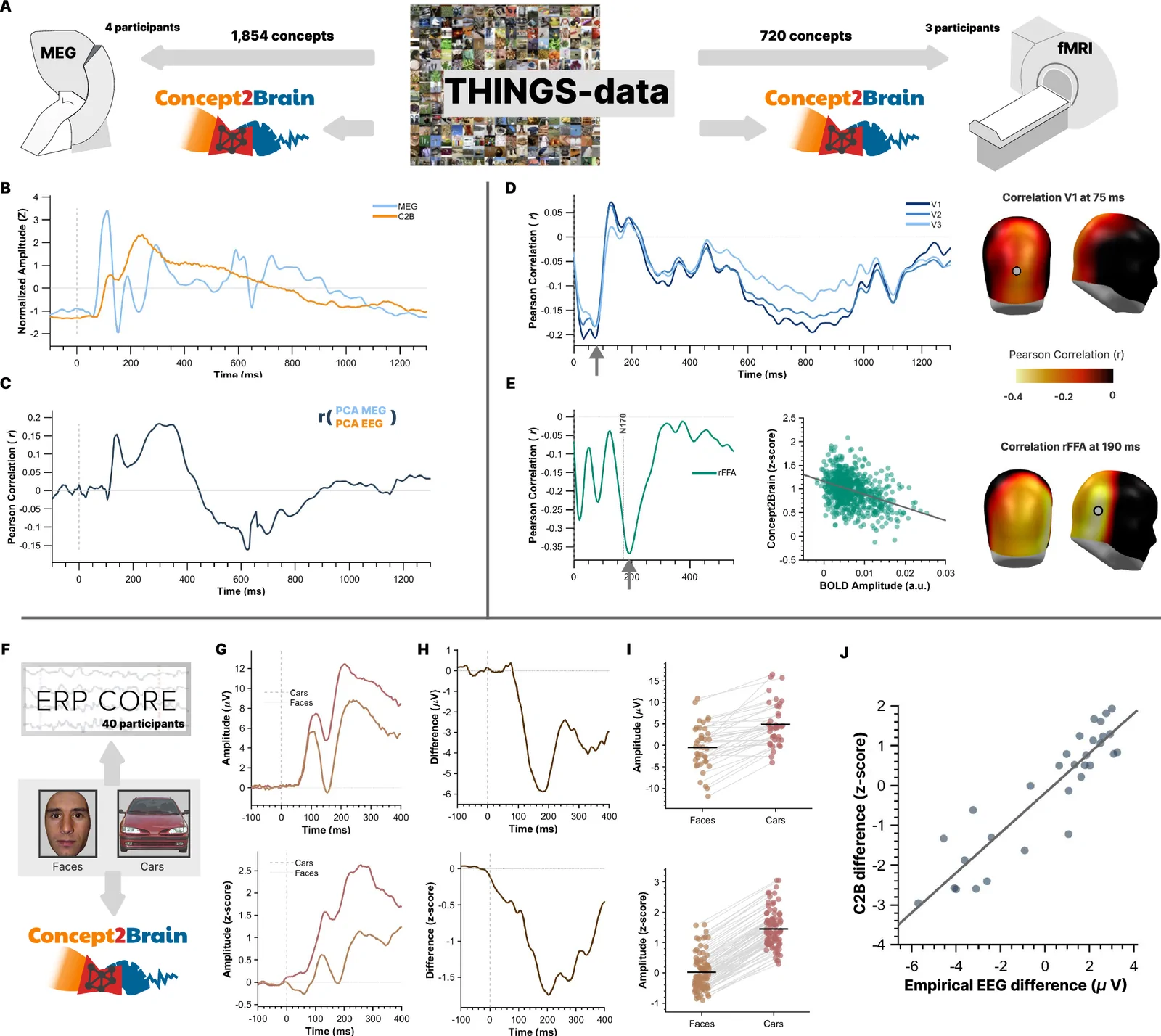
Out-of-sample validation using THINGS and ERP-CORE datasets. **A** Process of data production and validation using the THINGS-data. On the left, 4 participant’s brain responses to 1854 concepts were recorded using MEG. On the right, 3 participant’s responses to 720 concepts using fMRI were recorded. **B** Grand average of both MEG and Concept2Brain response at occipital sensors. **C** Two PCAs were computed to extract the major temporal component, one for each signal type, which were then correlated for each time point. **D** Bold activity from visual areas (V1, V2 and V3) was likewise extracted and correlated with the Concept2Brain activity for each time point (left). Maximum correlations were found at 75 ms, and this time was used to correlate BOLD and EEG activity across the sensor topography (right). **E** As with **D**, a comparison was performed but using the right fusiform face area (rFFA), involved in generating the face-sensitive N170 component. Here, maximum correlations were found at around 190 ms (left), and EEG activity at this time point was linearly related to BOLD activity from the rFFA (center). Again, this time point was used to correlate BOLD with the Concept2Brain outputs for each sensor, resulting in the expected right-lateralized topography, typical for face processing. **F** Process of validation with the ERP-CORE dataset, specifically using the data set with the face-sensitive N170 ERP component. **G** Grand average of ERPs evoked by faces and cars, for the ERP CORE (top) and Concept2Brain (bottom) from right-posterior electrode PO8. **H** Difference between faces and cars at the PO8 electrode, showing the modulation of the N170 component. **I** N170 amplitude differences between faces and cars for each participant, for empirical (top) and Concept2Brain data. Each point represent 40 participants of the ERP-CORE dataset (top), and 88 participants of the Concept2Brain output (bottom). **J** Scatter plot illustrating the topographical correspondence between face-specific responses at 190 ms from empirical (ERP-core) and Concept2Brain signals. Each dot represents one of the sensors shared between the EEG sensor arrays. The ERP-Core dataset is attributed to: Kappenman et al.^40^. ERP CORE: An open resource for human event-related potential research. NeuroImage, 225, 117465. It is provided under the CC-BY-SA 4.0 license (https://creativecommons.org/licenses/by-sa/4.0/). No changes to the ERP-core data were made in the present article.

The first dataset (THINGS-data) contains a large set of images involving 1,854 unique concepts^32,33^. These images were presented to human participants for 1.5 s and data collected using a variety of neuroimaging tools (see Fig. 4A). Four participants were recorded using magnetoencephalography (MEG) and three participants using functional magnetic resonance imaging (fMRI)^33^. Although this dataset was not originally recorded using EEG, it offers a suitable framework to compare the present model’s output with empirical out-of-sample data. Therefore, we fed the Concept2Brain model the same images used in both the MEG and fMRI experiments and compared their output.

First, we compared effects in THINGS-MEG recordings to corresponding effects as predicted by the Concept2Brain model for 1854 pictures (Fig. 4B). This was done by applying principal component analysis (PCA) and extracting the first principal component from both the MEG and EEG data, to allow comparisons between the two modalities. Then, a Pearson correlation coefficient was estimated between the empirical (MEG-PCA) and simulated (EEG-PCA) data for each time point across the 1,854 pictures. The highest correlation indices appeared during components corresponding to early visual processing (C1 component^34^) and subsequent semantic processing (P3 component). Furthermore, a substantial negative correlation was present during the LPP component, suggesting opposite sign in polarity but high resemblance of amplitude differences acriss the synthetic and empirical data.

Following the MEG comparison, we compared the THINGS-fMRI BOLD activity collected from 720 pictures with the EEG output of the Concept2Brain model (see Fig. 4D, E). To this end, we averaged the BOLD activity of the V1, V2 and V3 regions and correlated them with activity from Concept2Brain sensor Oz for each time point, across the data from 720 pictures (see Fig. 4D). Consistent with prior work^35,36^, we observed a high correlation at 75 ms in the V1 region (Pearson’s *r* = −0.206, *p* < 0.001), and slightly smaller correlations for V2 and V3, suggesting that Concept2Brain accurately reflects different visuocortical activation for each picture as measured by fMRI. It is noteworthy that the LPP window (400 ms to 800 ms) also exhibited a robust correlation between fMRI and Concept2Brain activity, in line with fMRI studies with complex scenes^37^.

Next, we repeated this analysis with THINGS-fMRI data from the right facial fusiform area (rFFA) and simulated data, with a focus on the right posterior electrode site PO8 where face responses in EEG signals are maximal (see Fig. 4E left). In this analysis, the expected maximum correlation between empirical BOLD in the rFFA and simulated EEG activity was found at around 190 ms (Pearson’s *r* = −0.367, *p* < 0.001), covering the prototypical time window of the face-sensitive, negative-going, N170 component of the ERP. Analyses across all sensors found that this expected negative relation with heightened BOLD predicting more negative EEG signals was maximal in the right posterior area (see Fig. 4E center). This aligns with previous findings of a strong relationship between activity in the rFFA and the amplitude of the N170 component^38,39^. Taken together, the validation using fMRI and MEG data from the THINGS-data set suggests the utility of the Concept2Brain model in capturing neural dynamics and its potential for generalizingconceptual information into synthetic datasets that display external validation properties of empirical EEG data.

The second out-of-sample dataset utilized to validate the Concept2Brain model is the N170 subset from the ERP CORE dataset^40^ (see Fig. 4F). This dataset contains data from 40 participants viewing 40 pictures of faces and 40 pictures of cars. As mentioned above, a substantial body of prior work^41,42^ has found differences between ERP signals evoked by faces, compared to non-faces, around 170–200 ms post-stimulus, at right posterior electrodes. Here, we use this effect to examine the predictive validity of the Concept2Brain output by feeding the stimuli from the ERP CORE dataset as input into the Concept2Brain model. Figure 4G shows the resulting grand average waveforms for faces and cars at the right-posterior electrode location PO8, showing distinct activity for each condition both in the empirical ERP CORE and synthetic Concept2Brain output. Across empirical and synthetic data, the difference between the conditions shows similar latency (see Fig. 4H) and morphology (see Fig. 4I). We quantified the similarity of the empirical and simulated data using Pearson correlations of the difference between conditions (faces minus cars) at 200 ms across EEG sensors. A high correlation (*r* = 0.91, *p* < 10^−11^, see Fig. 4J) was found, indicating robust prediction of the face-specific voltage difference topography by the Concept2Brain model.

### Modulation of electrophysiological data by emotional scene content

Next, we validated Concept2Brain against ground truth electrophysiological correlates of emotional processing. To assess the predictive validity of Concept2Brain with respect to electrophysiological signals related to emotional content, we conducted three analyses.

**Fig. 5:**
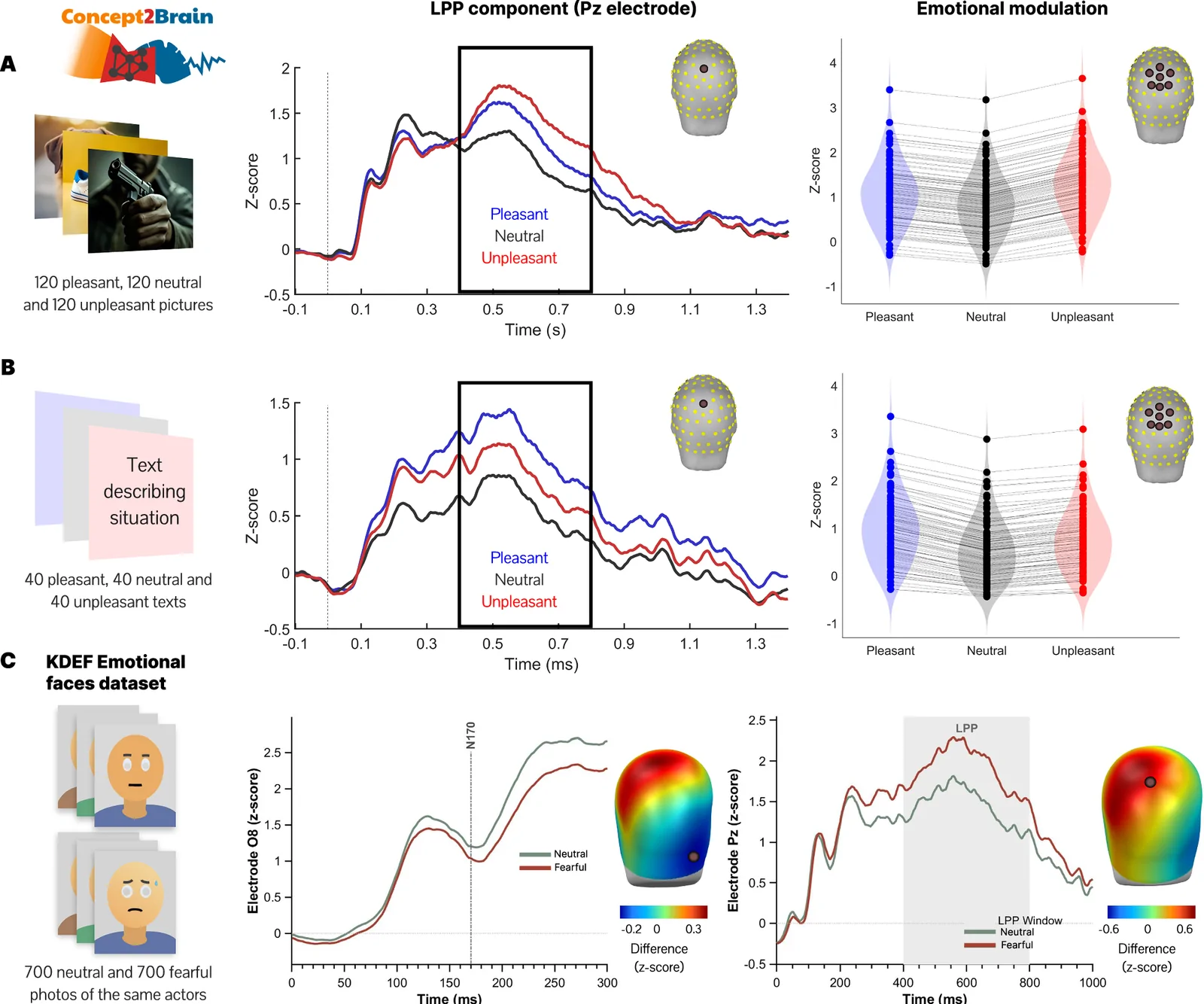
Validation of Concept2Brain with emotional scene content. **A** EEG data were generated using the same pictures employed for training the model, 120 pictures per emotional content category (pleasant, neutral, unpleasant). The central panel displays the temporal evolution of the LPP component, where emotional pictures prompt heightened amplitudes compared to neutral pictures. In the right panel, scatter plots indicate that this LPP effect is reproduced across all 88 synthetic participants. **B** The model was also tested with text stimuli describing emotional situations instead of pictures. Temporal dynamics again show a stronger LPP response for emotional texts than for neutral descriptions. **C** Two sets of 420 pictures depicting “fearful” and “neutral” expressions were fed into Concept2Brain. The middle panel presents a line plot and a topographic map, highlighting the well-established N170 effect reported in the literature on emotional face perception. The right panel displays a line plot and a topographic map, showing a higher LPP amplitude when viewing “fearful” compared to “neutral” expressions, over parietal sites. Stimuli were obtained from the Karolinska Directed Emotional Faces (KDEF)^53^ and corresponds with pictures of human participants showing emotional expresions.

First, we assigned the same pictures used for training the model (120 pleasant, 120 neutral, and 120 unpleasant pictures) as inputs to the trained model and compared the output waveforms as a function of emotion category (see Fig. 5A). Consistent with a large body of prior findings^19,26^, we observed emotion-related differences at centroparietal sensors between 400 and 800 ms post-onset, corresponding to the late positive potential (LPP; Fig. 5A center), known to be modulated by emotinla scene content. Emotional pictures prompted greater LPP voltage compared to neutral pictures (Fig. 5A right) and this effect was observed for each synthetic participant^19,26,43^.

One of the key features of the Concept2Brain model is its ability to extract conceptual latent representations from both pictures and texts. To test this, we fed the model with a total of 120 text stimuli, leveraging the multimodal capabilities of CLIP, and simulated EEG time series for each of the 88 virtual participants. Many studies have compared ERPs to complex, naturalistic pictures varying in emotional content^44,45^. Following standard procedures in this field, we divided the trials into pleasant, neutral and unpleasant exemplars based on the emotional content of the text input. Figure 5B, center, shows the ERP at mid-parietal electrode Pz. As expected, activity during the early time segment (from 0 to 200 ms) was similar between pleasant, neutral, and unpleasant categories, because this portion of the brain signal is defined by processes related to perceptual analysis, with little sensitivity to emotional content. However, after 300 ms, condition differences became more evident. In accordance with empirical findings^19,26^ (see training datasets in Methods section), emotionally engaging content (i.e., the pleasant and unpleasant conditions) prompted greater ERP amplitude than emotionally neutral content at parietal sensors from 400 to 800 ms post-onset (Fig. 5B right). All virtual participants showed the typically observed pattern with heightened LPP amplitudes for emotional stimuli^43^, indicating that the Concept2Brain model robustly reproduces typical empirical findings. It is important to note that the model was trained solely on picture-evoked EEG data as described above. Thus, text-prompted simulations remain less validated and should be treated with additional caution.

In a final step, we tested the sensitivity of Concept2Brain to emotional facial expressions. As stated above, prior research with emotional faces^46,47^ has shown a selective increase in the face-selective N170 ERP component, observed over right-occipital sites, when viewing emotional faces as opposed to neutral faces. Additionally, the LPP component described above has also been shown to be modulated by emotional facial expressions, with a heightened response to emotional faces^47^. To validate Concept2Brain, EEG waveforms were generated using exemplars from the Karolinska Directed Emotional Faces (KDEF) set as input. A total of 420 fearful faces and neutral faces from 140 different people were used (see Fig. 5C left). Exemplars contained the same actors and the same background for each expression, to control for non-emotion differences. Figure 5C, center panel, shows a larger N170 response in response to fearful compared to neutral faces. as expected, this effect is mainly seen at right posterior sensors. The LPP modulation, as shown in Fig. 5C right, also showed a higher neural response for fearful faces, compared to neutral. This mirrors empirical studies with the same stimuli, supporting the predictive validity of the Concept2Brain model.

### ERP generation: web implementation

**Fig. 6:**
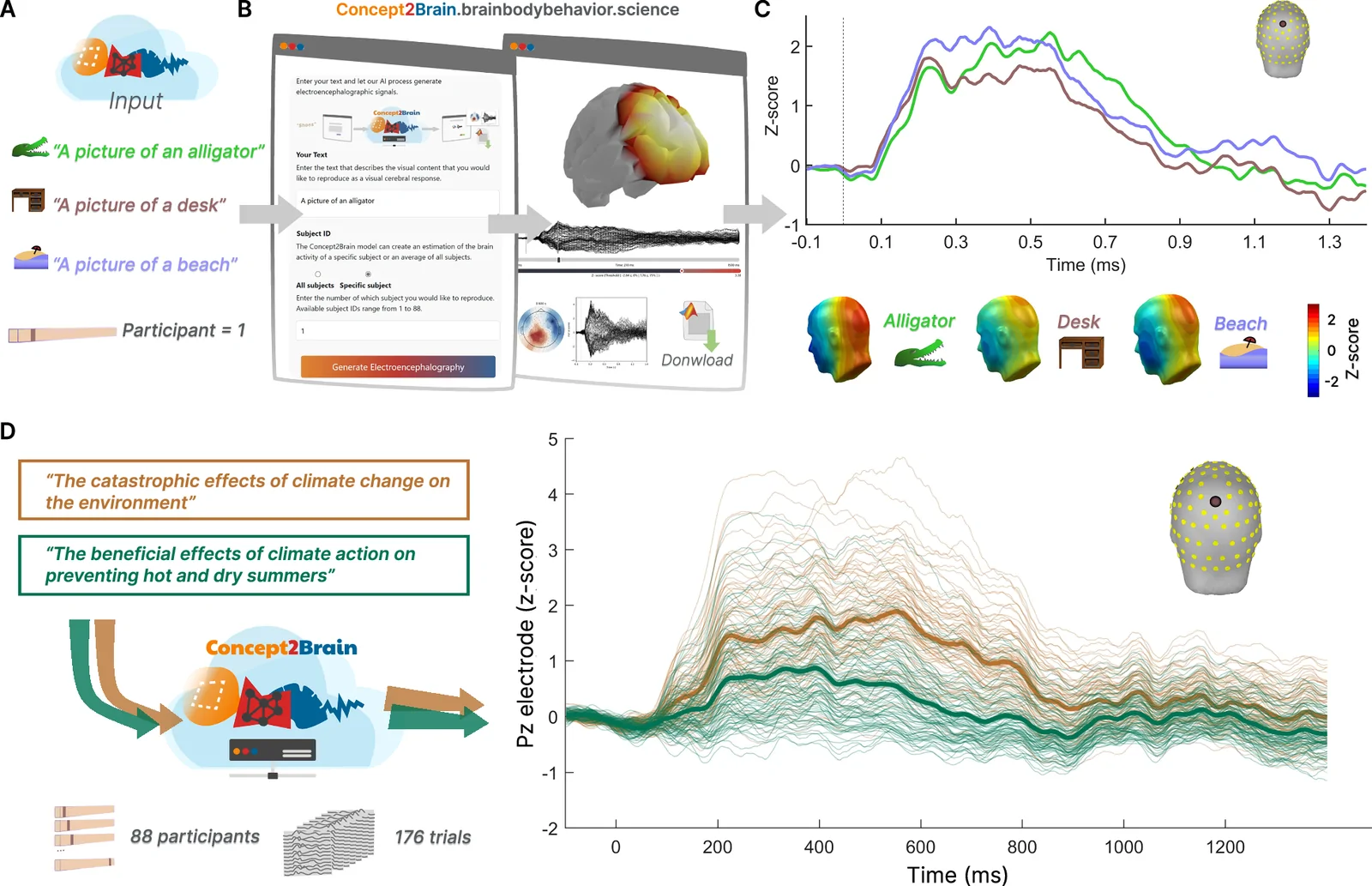
Workflow for generating individual or full-sample datasets for pictures or written texts using the cloud-computing platform of Concept2Brain. **A** In the first example, simple written texts were entered into the model such as “a picture of an alligator”. In this initial example, only data for the first virtual participant were generated. **B** The web platform offers an interface for uploading a picture or a written text input. Once the information is submitted, the platform generates the synthetic ERP signal. Then, the resulting data are displayed in an interactive 3D brain model across the time series along with a 2D projection, viewed from the top. The generated ERP data are readily downloaded in the MATLAB.mat format, used widely for EEG/ERP post-processing. **C** These data are exemplified by the ERP waveform at electrode Pz for virtual participant number 1, showing synthetic ERPs in response to text indicating pictures of an alligator (green), a desk (brown), and a beach (blue). In the bottom row, the topography of such generated ERPs is shown for the time window 400 ms to 800 ms, illustrating how neutral content such as a desk prompts lower late ERP responses than emotionally engaging content. **D** As a second example, the Concept2Brain model can also represent how 88 virtual participants respond to content related to more complex issues, difficult to implement in an empirical study. This dataset reflects two written texts describing complex content of interest and thus consists of a total of 2 × 88 = 176 trials.

We also tested the cloud computing web interface of Concept2Brain by synthesizing EEG datasets in response to individual inputs. An empirical study would require the use of dozens of repeated trials using the same picture or text in order to obtain a clean averaged waveform. However, the Concept2Brain model allows researchers to estimate the averaged waveform associated with this concept in one single step for one virtual participant. As an example, we tested three concepts related to unpleasant (alligator), neutral (desk) and pleasant (beach) emotional content. These texts were fed into the model and the synthetic ERP responses are shown in Fig. 6A–C).

As noted previously, emotional content prompts greater ERP amplitudes during the LPP time window^19,43^ (see Fig. 6C). Here, the model produced a participant-level time series that reproduces this common finding. Using the Concept2Brain platform, these patterns of response can be examined at the level of individual prompts and individual participants, without the need of large datasets. The platform can be accessed at concept2brain.brainbodybehavior.science and generated data can be downloaded in the MATLAB file format (.mat), a widely used platform in EEG/ERP research^48^.

Finally, for illustration, we offer an example of how the platform may aid in producing synthetic data for researchers interested in complex semantic content. Such content is often difficult to study empirically, requiring extensive stimulus curation and control, while prompting substantial inter-individual differences. In Fig. 6D we illustrate the usage of the Concept2Brain approach for simulating brain responses to two abstract statements about climate change. The model was used to simulate the neural response of 88 virtual participants. The synthetic data indicate heightened brain responses to statements that emphasize aversive consequences rather than statements mentioning mitigation strategies (Fig. 6D, right panel). Again, these findings are consistent with empirical studies into higher-order cognitive processes such as negative affective biases^49^.

## Discussion

Electroencephalography is arguably the most widely employed measure of human brain activity. Its sensitivity to neural processes in real time renders it a powerful tool used in a variety of disciplines including psychology, medicine, economics, educational technology, in addition to neural sciences. Here, we introduce the Concept2Brain model, a computational architecture for converting information contained in text or pictures into synthetic ERP waveforms—representative brain electric responses to events.

The architecture consists of a cross-domain conditioned Variational Autoencoder^50–52^ algorithm connecting conceptual information with brain responses. It is trained on more than 10,000 trials with 360 complex naturalistic pictures, representing a wide range of content. Cross-validation with a held-out set of pictures showed robust generalization of the model to new input. Data reconstruction analysis showed high correlations between the empirical ERP signal and the synthetic signal produced for the same pictures. The highest correspondence was found in central parietal regions between 400 ms and 800 ms, where the topography and latency matched the LPP component, a brain response modulated by a range of attentional and emotional processes when viewing complex visual stimuli^19,26,43^. Synthetic waveforms showed different electrophysiological responses for each participant, indicating expected inter-individual differences between virtual participants^28–30^, while also robustly reproducing major prototypical ERP components across the synthetic sample. This finding included an accurate reproduction of the well-established modulation of the emotion-sensitive LPP component^19,43^ when manipulating emotional content of the input.

Out-of-sample validation of the model with data from two publicly available data sets likewise demonstrated correspondence between synthetic and empirical data. First, recordings from the THINGS-data^33^ multimodal dataset were correlated with the Concept2Brain synthetic output using the same concepts. Empirical hemodynamic responses to pictures of various objects in visual areas such as V1, V2, V3 and rFFA showed substantial correspondence with object-related differences in synthetic EEG generated by Concept2Brain, although these associations were weaker than in the cross-validation analyses within the orginal data set. Similarly, we employed the systematically validated ERP CORE^40^ dataset to offer a direct comparison between empirical data and the corresponding Concept2Brain output. The empirical ERP CORE data collected during a face viewing task and the Concept2Brain output with the same stimuli showed significant correspondence overall, while again these associations were weaker than in the original data set. Specifically, Concept2Brain accurately reproduced the well-documented face-selective modulation of the right-posterior N170 component. Taken together, Concept2Brain allows estimation of cortical activity even when using distinct brain recording systems and different paradigms. Users should be careful to not overinterpret simulated data, especially across different recording systems, paradigms, and stimulus types.

The Concept2Brain model showed additional generalization abilities outside the training set: For example, when generating EEG activity from 1078 standardized affective pictures, distinct from those used to train the model, the amplitudes of the synthetic LPP component showed a robust correlation with the normative arousal ratings for each picture. When fed with facial expressions from the KDEF picture dataset^53^ the Concept2Brain model captured known differences between fearful and neutral facial expressions. It is important to note that these effects were never introduced during training; they were instead learned implicitly from the data. Although the cautionary note above applies to these observatinos as well, these results highlight the potential of deep learning methods to abstract subtle and complex features of electrophysiological dynamics from EEG data. Future versions of Concept2Brain may incorporate additional data, heightening the precision and generalizability of its predictions.

Currently, the architecture relies on the CLIP architecture developed by OpenAI^54^. This model allows researchers to work with cross-modal inputs, enabling them to employ both pictures and text. This is helpful when pictures are not available, or when content cannot be graphically represented. However, the Concept2Brain model generates brain responses to visual pictures, while text prompts may not precisely correspond with any given picture. As a consequence, the use of text prompts instead of actual pictures may lead to more variable and noisy predictions. Therefore, users feeding the model with text prompts should be particularly cautious and seek additional converging evidence, e.g., from published studies in their field of interest. In addition, with ongoing rapid advancements in AI development^55–57^, CLIP may not represent the most up-to-date interpreter model. The Concept2Brain methodology does not depend on this specific module, and is set up to readily allow usage of newer, more capable, encoders in future versions.

Currently, there are several brain encoder models using non-linear AI-based processes to estimate various types of brain responses to stimuli^58–60^. For example, Benchetrit et al.^59^ also used CLIP and autoencoders to decode time-varying brain responses. However, to our knowledge, the Concept2Brain model is the first to systematically generate EEG data from synthetic human participants. In contrast to just predicting an averaged brain response like other models^58–60^, the Concept2Brain model provides a framework and web-based platform to simulate new datasets.

**Fig. 7:**
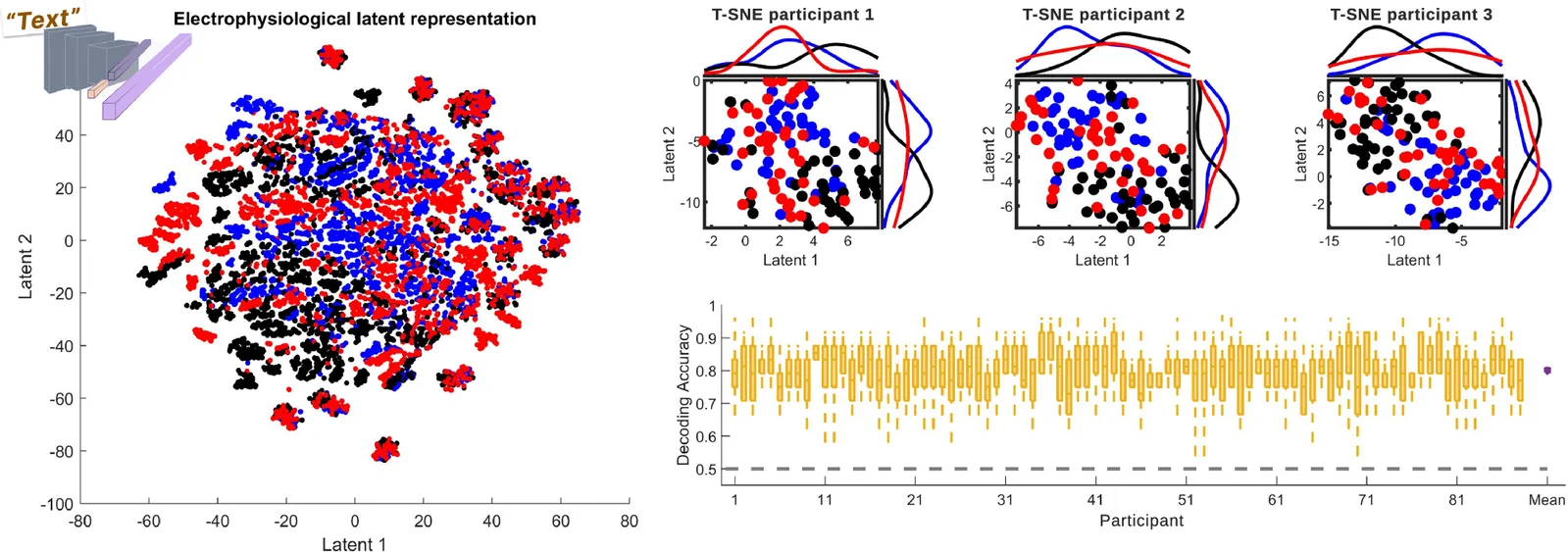
Electrophysiological latent space for affective texts. Left: Scatter plot of the electrophysiological latent space from all affective written texts projected into two dimensions, illustrated using the T-SNE method. Blue, black and red colors represent single trials of pleasant, neutral and unpleasant affective texts from 88 participants. Right top: Electrophysiological latent space for all written texts in the first 3 participants. Plots show how emotional content (pleasant and unpleasant) can be distinguished from neutral content. Right bottom: A three-class support vector machine classifier was applied to the electrophysiological latent space for each synthetic participant, demonstrating high discrimination between the three affective text categories for all synthetic participants. Each yellow boxplot represents the classifier model accuracy of ten random splits for each participant. The center line represents the median, while the bounds of the box represent the first quartile (25th percentile) and third quartile (75th percentile). The purple point represents the grand average across participants.

AI architectures like the variational autoencoder^52,61,62^, at the core of the Concept2Brain model, represent a recent advance in signal processing^63,64^. In contrast with other bio-realistic models like the Jansen-Rit algorithm, spiking neural networks^65^, or the human neurocortical neurosolver (HNN) model^66^, the present autoencoder architecture does not focus on neurophysiological mechanisms but on reproducing prototypical signal waveforms and topographies that are characteristic for a given input. Similar approaches are increasingly applied in neuroscience research and may complement biologically motivated models. For example, Abdi-Sargezeh and colleagues used a VAE architecture to define a neurophysiological latent space from which intracortical activity can be generated^64^. In the present study, the cVAE captures electrophysiological features and compresses them into 256 normal-distributed variables. This electrophysiological latent space captures and normalizes the variability in the brain data, modeling a low-noise representation of the brain response to a given input (consult the Supplementary Information SI1 for more information on how electrophysiological representations are encoded in the latent space). Figure 7 shows the electrophysiological latent representation of 10,560 affective written texts obtained from the 88 virtual participants (see Results section 3.2).

The left panel of Fig. 7 displays all trials from all participants, illustrating the autoencoder’s ability to capture inter-individual variability in neural response patterns^28,29^. Importantly, emotion-specific differences remain evident at the individual participant level (top right panel). This autoencoder-based refinement of the electrophysiological signal enables highly accurate decoding of affective categories, as demonstrated by the support vector machine (SVM) classification results shown in the lower right panel of Fig. 7.

One limitation of the present model is the limited concept space, which is based on a sample of 360 pictures. Thus, the Concept2Brain model represents not a final product, but an evolving approach to connecting state-of-the-art AI models with neuroimaging outcomes^4,67^. Future iterations of the architecture will include new image encoding models, larger datasets, and responses to additional stimuli and tasks. The present cross-validation analyses however suggest that in its present form, the architecture is already useful for data generation and simulation studies. Through Bayesian updating, the VAE architecture learns to capture statistical structures in the training data. As a consequence, the model benefits from exposure to variability across participants and pictures, which results in high-fidelity model predictions even when relatively few trials are available. In contrast to other approaches that involve few participants viewing larger amounts of pictures, the datasets used here involve (1) significantly larger number of participants and (2) usage of standardized stimulus material, selected to cover a wide range of content in balanced fashion. This allows Concept2Brain to generate new datasets with up to 88 different synthetic participants while preserving a high degree of generalizability.

Potential uses of the Concept2Brain model span many disciplines. For example, the tool may contribute to modeling consumer behavior by illustrating predicted brain responses to marketing-related content^2^. In basic science contexts, the generation of synthetic EEG datasets will allow the estimation of variability and effect sizes of ERP data^28^. Finally, the Concept2Brain model provides an avenue for generating synthetic brain waves in response to any pictorial or text input^30^ and thus may be used to provide initial simulations of responses to new classes of stimuli. With its potential to be further developed and expanded, the proposed approach is poised to facilitate reproducible and open simulations of brain responses to a wide range of stimuli. Therefore, we deployed Concept2Brain as a cloud-computing service (concept2brain.brainbodybehavior.science) where users can utilize the model without any installation requirement. The web-based service enhances accessibility and maximizes software- and hardware independence along with ensuring that users benefit from future updates of the model.

## Methods

The Concept2Brain model consists of a deep neural network architecture trained to generate simulated electrophysiological signals in response to a wide range of semantic and emotional content. The model has been trained using EEG data from 88 human observers, viewing several hundreds of naturalistic scenes varying in semantic and emotional content. In the following sections, we give a detailed description of the deep learning architecture, its training, and its performance evaluation.

### Concept2Brain: a cross-domain conditioned variational autoencoder model

The Concept2Brain model comprises a cross-domain conditioned variational autoencoder architecture based on deep learning networks. This architecture can be divided into two main components, performing three tasks: (1) The first component is a conditioned variational autoencoder (cVAE)^50–52^, which is a neural network designed to extract latent dimensions from the electrophysiological data and reconstruct the original data from the resulting low dimensional latent space. (2) The second component is a cross-domain deep network, which is a dense multi-layer network translating information contained in the conceptual domain to the electrophysiological domain. This network converts pictures or text elements captured by an AI solution into a single vector embedding and then translates this concept-level latent information into the latent electrophysiological space of the cVAE. (3) Finally, both deep learning architectures are connected in the Concept2Brain model, converting picture or text information into simulated EEG recordings. In the following, we outline the properties of the components of our model.

### The conditioned variational autoencoder model for compressing and predicting electrophysiological data

The cVAE is a type of autoencoder (AE)^61,68,69^, a deep neural network in which the output data mirror the input data as closely as possible. Importantly, in the center of the AE architecture, hidden network layers converge into a smaller layer called the “bottleneck”. This low-dimensional layer is also referred to as the “latent space” and represents a compressed version of the data that contains a sufficient amount of information needed for the reconstruction of the input data (Supplementary Fig. 1.1, light purple vector). In the present cVAE, both input and output data consist of spatio-temporal matrices of ERP signals having a size of 129 (recording channels) by 750 (time points, representing 1.5 s at the sampling frequency of 500 Hz). These data are passed through an encoder, bottleneck, and decoder. Both the encoder and decoder architectures have three convolutional layers^70,71^, designed to extract spatial and temporal features from input data. In EEG and ERP analysis, convolutional layers are particularly useful for capturing the temporal dynamics of brain signals^72,73^. The convolutional layers of the Concept2Brain model extract the electrophysiological latent space, effectively compressing the spatio-temporal information of the EEG signal.

Unlike a standard AE, which learns a low-dimensional latent representation for input data but lacks a well-structured latent space for meaningful sampling, the cVAE model enables the synthesis of new data samples, the cVAE model enables the synthesis of new data samples^50^. Specifically, a standard AE encodes data into a bottleneck layer and reconstructs it without enforcing any distributional constraints on the latent space. As a result, the latent space may be sparsely populated and discontinuous, making it difficult to interpolate or sample new data points that resemble the training distribution—in the present case EEG data. In contrast, the variational AE (VAE), explicitly models the latent space as a structured distribution (typically Gaussian) conditioned on auxiliary information, ensuring that samples drawn from the latent space can be decoded into realistic and semantically consistent data—potentially reflecting biologically plausible features in domain-specific applications^50,74^. In the VAE architecture, the bottleneck contains two parallel layers that output the mean (μ) and standard deviation (σ) of the latent variable distribution. During training, a latent vector z is sampled using the reparameterization trick: $z=\mu+\sigma \cdot \epsilon {z}$ where $\epsilon \sim N\left(0,I\right)$. This approach enables differentiable sampling and ensures that points drawn from the latent space correspond to realistic reconstructions, thereby facilitating meaningful data generation. As a result, the latent dimension becomes interpretable, because it describes features in the electrophysiological space. Here, we used a size of 256 units for the bottleneck (both μ and σ layers). This value was chosen after preliminary tests with different bottleneck sizes to enable the representation of the variability across participants in the empirical training data. As a result, the VAE architecture enables the generation of new data by introducing 256 z-score values at the beginning of the decoder component of the VAE network and forwarding them through the architecture, until the output layer (see Supplementary Fig. 1.1).

The architecture also represents variability across participants. To this end, the cVAE incorporates additional information (conditioning variables) immediately before and after the bottleneck (Supplementary Fig. 1.1, light orange)^51^. In our architecture, the conditioned information represents a participant to whom the EEG input belongs. Thus, a vector of *n* x 1 (in our example, the total *n* is 88 participants) is added with a value of 1 in the index of the participant (one-hot-vector) and all other values set to zeros. This vector is added to the model just before and after the bottleneck layers (i.e., the latent vectors). By including this information, the cVAE forces the bottleneck to learn general electrophysiological properties by compressing the EEG signals regardless of the idiosyncratic properties specifically related to each participant. Then, the conditioning of the cVAE model allows the generation of new synthetic EEG recordings for each of the 88 participants reproducing some of their idiosyncratic electrophysiological responses. Thus, it is possible to effectively create simulated multi-participant studies, illustrating how different brains would respond to the input picture or text provided.

### The cross-domain bridge between the concept-level and electrophysiological latent spaces

The Concept2Brain model provides a link between electrophysiological information derived from cortical activity and concept-level content. To this end, the model relies on a third-party AI solution for extracting the conceptual representation of real-world stimuli such as pictures, texts, or audio. Currently, AI solutions and alternatives are rapidly evolving and increasing. Crucially, the Concept2Brain idea does not rely solely on one specific model, and its future versions may adopt different models as they advance or offer improved features related to diverse objectives.

One of these AI solutions is the Contrastive Language-Image Pre-Training (CLIP) model^54^ (Supplementary Fig. 1.2). We selected this model developed by OpenAI (accessible from https://github.com/openai/CLIP) because it captures the representations of images^56^ and texts^57^ from large-scale datasets and embeds them in a common latent space. Specifically, the CLIP model compresses the information contained in the input (pictures or text) into a multimodal vector of 512 dimensions. In Concept2Brain, this representation is called the conceptual latent space.

To connect both the conceptual representational space, obtained from CLIP, and the electrophysiological representational space extracted using our EEG-cVAE architecture, we applied a cross-domain multi-layer neural network^75,76^ (Supplementary Fig. 1.2, shaded network between top and middle rows). This part of the architecture consists of three dense fully connected layers of neurons. It abstracts non-linear information from the 512-size CLIP output and estimates the 256-size latent vector of the cVAE, thus mapping the conceptual information in the electrophysiological representational space. This architecture, comprising only three layers, was chosen to maintain model simplicity, to reduce overfitting, and to promote generalization.

### The Concept2Brain architecture: the cVAE and cross-domain neural networks

Finally, once both the cVAE (Supplementary Fig. 1.1) and then the cross-domain (Supplementary Fig. 1.2) models have been trained, the Concept2Brain model makes use of both models to generate new EEG recordings from concepts not previously presented to the human participants (Supplementary Fig. 1.3). Specifically, the workflow of the model starts by feeding one image or text into the CLIP model (Supplementary Fig. 1.3 left), which calculates one 512 multidimensional vector mapping the concept representation. Next, the cross-domain network translates this conceptual representation for one participant into the electrophysiological latent space(Supplementary Fig. 1.3 middle). Then, the decoder module of the cVAE takes this  μ vector information and the one-hot 88 vector indicating the participant identification to reconstruct the EEG signal (Supplementary Fig. 1.3 right). Following this workflow, the Concept2Brain model can encode any picture or text semantic and emotional content into a representation of brain activity.

A deeper understanding of the deep neural design and parameters of the model can be obtained through the open-source script accessible from https://github.com/ASantosMayo/Concept2Brain. In addition, interpretability analyses can be found in the Supplementary Information (SI1) explaining how electrophysiological representations are encoded in the latent space.

### Training the Concept2Brain model

In the following section, we describe (1) the empirical data derived from human EEG recordings used to (2) fit the cVAE model to learn the electrophysiological properties of data. Then, (3) the cross-domain model is trained based on the CLIP output and the cVAE latent space.

### EEG datasets

The Concept2Brain model aims to reproduce prototypical electrophysiological brain responses evoked by visual input. To achieve this, two EEG datasets from studies employing passive viewing tasks were utilized. In both studies, a total of 88 participants viewed a wide range of naturalistic photographs that can be categorized into three broad content types: pleasant, neutral, and unpleasant.

**Fig. 8:**
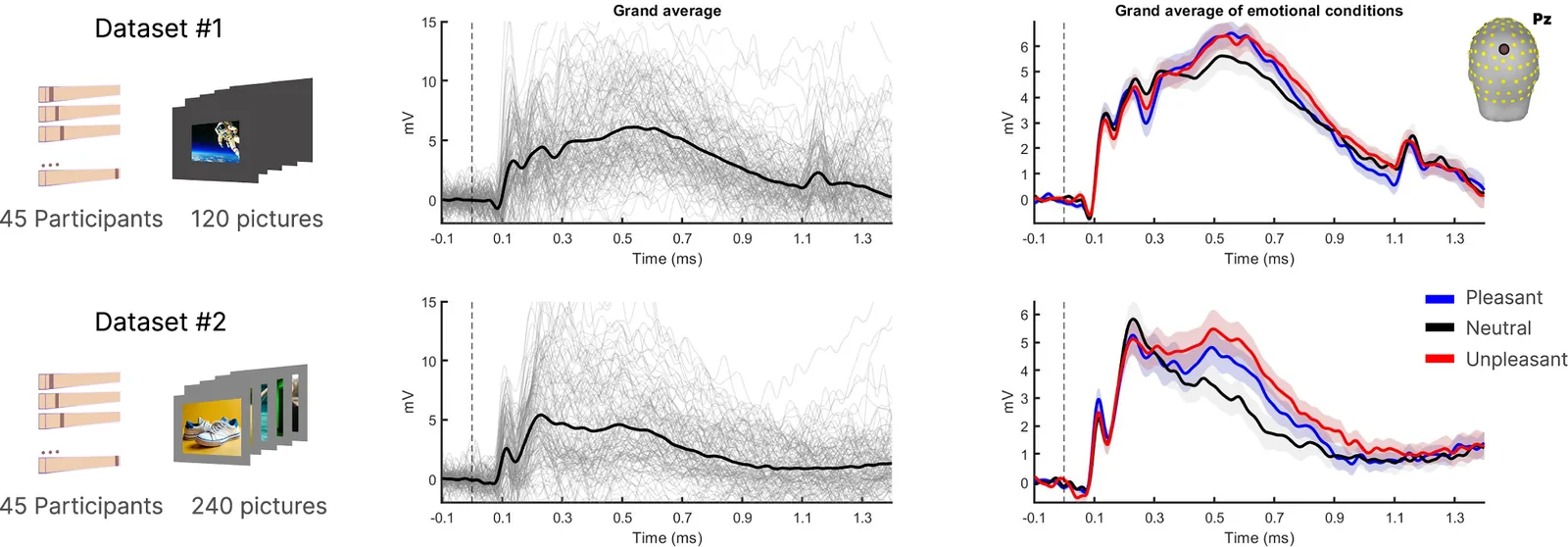
Empirical EEG datasets used for training the Concep2Brain model. Differences between both datasets illustrate differences in the presentation mode (left column): Dataset #1 involved displaying a smaller picture on a dark background (top) and Dataset #2 involved displaying a larger picture on a gray background (bottom). The middle column shows the grand average of picture-evoked ERPs, while the right column shows the averaged evoked responses by affective category (blue, red and black colors refer to pleasant, neutral, unpleasant conditions, respectively). Shaded areas around the line plots in the right column represent the standard error of the mean (SEM) for each condition.

Dataset 1 (Fig. 8 top) represents the EEG recordings of 45 participants watching a total of 120 pictures (40 pleasant, 40 neutral, 40 unpleasant). All participants viewed the same set of pictures twice, presented in a randomized order that differed across participants. In this experiment (Dataset #1), pictures were shown during 1 s as a rectangular shape (spanning 9° of visual angle horizontally) at the center of a computer screen over a black background. The inter-trial interval (ITI) was jittered randomly between 2 s and 4 s. Dataset 2 (Fig. 8 bottom) contains the EEG recordings of 43 participants, viewing a total of 240 pictures (80 pleasant, 80 neutral, 80 unpleasant). Participants viewed each picture once, the presentation order being randomized individually for each observer. For Dataset 2, a gray (50% luminance) background was presented throughout the experiment and pictures were shown during 2 s at the center of the screen, but spanning 15° of visual angle horizontally, with a jittered ITI from 4 to 7 s. Participants received course credits as compensation, and the study was approved by the University of Florida’s institutional review board, in accordance with the Declaration of Helsinki. Pictures for both studies were taken from the International Affective Picture System (IAPS)^77^ and other sources, including prior published work from the University of Georgia^78^. Categorization of each picture into pleasant, neutral and unpleasant conditions was defined according to affective ratings provided in the IAPS database and average participant ratings from prior studies^78^. Each study featured a separate set of pictures, resulting in a total of 360 unique pictures across the datasets. The use of two datasets with different presentation parameters (background and foreground color, edges, and visual degrees) was intended to facilitate generalization of conceptual picture content over specific visual features.

EEG was recorded using a 128-channel HydroCel net with Ag-AgCl electrodes (impedances below 60 kΩ), connected to an EGI Net Amps 300 amplifier (sampling rate of 500 Hz). The vertex electrode (Cz) served as reference. Data were preprocessed offline, filtered using Butterworth filters with cut-off frequencies of 0.2 and 25 Hz and filter orders of 3 and 9, respectively. Then, EEG data were segmented 0.1 s pre-event and 1.4 s post-event, resulting in trials of 1.5 s (750 time points). The Statistical Control of Artifacts in Dense Array EEG/MEG Studies (SCADS^79^) was used for artifact rejection. This method uses a quality index of the median absolute voltage, standard deviation, and maximum derivative of voltage to reject bad trials showing signal artifacts, and to identify bad EEG channels. Those channels were then interpolated using spherical spline functions^79^. After the SCADS procedure, data were mathematically transformed into a common (average) reference space and the mean of a pre-stimulus baseline of 100 ms was subtracted from each trial, for each channel. Finally, data were z-transformed before entering the cVAE (see below) to facilitate smoother convergence during training. A total of 13,640 artifact-free EEG signal trials derived from 88 participants (across both datasets) were used to train the model. A detailed description of samples and EEG pre-processing protocol is given in Gilbert et al.^80^.

### Training of the cVAE model to abstract electrophysiological features

The cVAE architecture was designed using the Python-based library Pytorch^81^ (version 2.4.0). It consists of one encoder, decoder, and bottleneck blocks. Both encoder and decoder blocks are formed by 3 convolutional layers (all using a kernel size 3×3, stride 2, padding 1), with a Rectified Linear Unit (RELU) activation function, commonly used to produce sparsity and abstraction of non-linear information from data^82^. The shape of autoencoder input and output data was identical: 129 EEG channels by 750 time points, representing −0.1 s to 1.4 s (frequency sampled at 500 Hz). Afterwards, the convolutional output from the encoder layers was flattened, resulting in a 204,544-dimensional vector representing the spatio-temporal properties of the EEG signal. To include the participant information (conditioned element of the cVAE), this vector was concatenated with another vector of size 88 containing zeros and a one in the index of the participant (one-hot vector).

This information was condensed by two fully connected layers, reducing the concatenated vector described above into two 256-dimensional vectors (μ and σ). Thus, the EEG signal was compressed and forced to follow a Gaussian-shape distribution where μ assumed values from N(0,1), and a complementary standard deviation was generated as the σ vector. This was implemented using the “reparametrization trick”^50^, an algorithm that samples input data into this Gaussian distribution (see below). Next, the 256-dimensional μ was concatenated again with the participant vector of size 88. Mirroring the encoder, this information was converted to a 204,544-dimensional vector through two fully connected layers and finally was passed into 3 convolutional layers to generate the 129 channels by 750 timepoints output matrix (i.e., the synthetic EEG data).

For training, the reconstruction loss must be calculated. It represents the difference between the actual data (the empirical EEG signal) and the output of the neural network (the synthetic EEG signal). Here, the Mean Squared Error (MSE) was employed because both data are continuous and the MSE is a simple and widely used index of loss. However, this reconstruction loss alone cannot ensure the correct learning of the μ and σ values, i.e., the VAE functioning. Thus, the Kullback-Leibler (KL)^74^ divergence was also estimated to facilitate the “reparametrization trick” and to ensure Gaussian distributions, using the following equation:

1 $${{KL}}_{{loss}}=\,-\frac{1}{2}\sum (1+\log \left({\sigma }^{2}\right)-\,{\mu }^{2}-\,{\sigma }^{2})\,$$

This loss index indicates how μ (the posterior distribution) deviates from the normal distribution (the prior distribution). Therefore, to force the neural network to approximate the Gaussian distribution of the μ vector to follow, the KL loss is summed together with the reconstruction loss indexed by the MSE. This simple addition of the KL loss at the beginning of the training however prevents the neural network from deeply exploring data to find the best reconstruction path. Therefore, an “annealing” strategy^83^ is commonly used, where the KL loss is gradually included throughout the training epochs, thus leaving the training to obtain the best reconstruction indices before starting to force the Gaussian distribution of the latent space. Here, we added an increasing KL loss across the training phase using the following sigmoid function:

2 $${{KL}}_{{epoch}}=\beta \cdot \frac{1}{1+{e}^{(-0.5\cdot \left({{{\rm{epoch}}}}-100\right))}\,}$$

where β corresponds to 1 × 10^−5^. This parameter is needed to scale the KL loss with respect to the reconstruction loss and prevents the “posterior collapse”^74^ effect. When it occurs, μ values approach 0, resulting in a skewed distribution instead of a Gaussian-like distribution and attenuating the generative capabilities of the model.

The training phase (Adam optimizer, learning rate 1 × 10^−6^) of the cVAE was performed with a random selection of 80% of the EEG data (10,912 trials, batch size 64). A total of 1300 epochs were needed to fully train the model and obtain the electrophysiological representation of 10,912 EEG trials (randomly selected 80% of total data). A multi-GPU machine containing 3 NVIDIA RTX 4070 SUPER Ti (16 Gb vRAM) was employed. Due to model weight, encoder, bottleneck and decoder components of the cVAE were allocated in each GPU and training dataset driven for each component and GPU.

### Inference of the conceptual space and training of the cross-domain network

To create the cross-domain model, a latent vector containing both conceptual and electrophysiological representations is required. In a first step, the CLIP model was employed to generate a 512-sized embedding for each of the 360 affective pictures viewed by the participants in the two EEG experiments. Second, the 10,912 EEG trials were passed into the cVAE model, extracting the 256-size vector μ. This resulted in a training data set consisting of 10,912 inputs of 512 + 88 values (conceptual information) derived from the pictures and an output of 256 values.

The cross-domain neural network was composed of 3 fully-connected dense layers. The first layer input size was 600 (512 CLIP embedding + 88 participant condition one-hot-vector), with an output of 256 neurons. Subsequently, the second layer converted the 256-size input to a 512-size output. Finally, the third layer reduced the output to 256 elements, matching the dimension of the electrophysiological latent space. This expansion-compression strategy was intended to prevent overfitting and enhance the generalization of the Concept2Brain model while maintaining participant-level information. The first two layers used an exponential linear unit (ELU)^84^ activation function. Again, an Adam optimizer^85^ (learning rate1⋅10^−3^) and MSE loss informed the training. The training phase was completed within 10 epochs, when the validation loss indicated that it had reached the best model parameter estimation, while ensuring generalization (i.e., avoiding overfitting).

### Evaluating the Concept2Brain model

Upon competition of training, the Concept2Brain model was able to generate new EEG signals based on pictures or texts (Supplementary Fig. 1.3). To measure model performance and capabilities, four main analyses were carried out:

First, we assessed model validity by evaluating its generalizability via 10-fold held-out cross-validation. For this analysis, we trained the model using 80% and 10% of the pictures as training and evaluation data, i.e., the EEG data from 288 and 36 pictures, respectively. Thus, EEG activity from 10% of the pictures (36) were held out of the training process and never presented to the architecture, serving as validation set. After the training phase, these validation pictures were fed into the model to generate the synthetic EEG activity from each synthetic participant. We simulated only artifact-free trials from participants and pictures that were contained in the original data set. As a result, the corresponding trials from empirical participants were reproduced, allowing a direct comparison between matching synthetic and out-of-sample data. Next, Pearson’s correlation coefficients were calculated to measure the similarity between empirical and synthetic signal across time points, channels and pictures. This procedure was repeated 10 times (folds), while exchanging the pictures falling in the training, evaluation and validation sets. As a result, the generalization capability of the model was measured for each one of the 360 pictures.

Second, data recovery was quantified by inputting the 360 pictures from the original training set into the Concept2Brain model. Thus, a large synthetic dataset was created, consisting of 88 pseudo-participants and 360 pictures composed summing a total of 31,680 ERP trials. Pearson’s correlations for each channel and time point were computed between empirical and synthetic averages. In addition, we assessed correspondence between empirical and synthetic data in the time-frequency domain. To this end, time-frequency analysis was carried out in a subset (data set 1) of empirical and synthetic data.

Third, the validity of the Concept2Brain model was assessed using other data sets, not seen during training. Stimuli from THINGS^33^, a widely known dataset containing 4 MEG and 3 fMRI participants viewing pictures depicting 1854 and 720 objects^32^ respectively were fed into Concept2Brain, yielding a synthetic EEG response for each object. Since the synthetic EEG activity and the MEG measurements do not share the same physical and source characteristics, a principal component analysis (PCA) was computed over the datasets in order to extract the visual components from the data structures. Next, we used Pearson’s correlation to measure the similarities between both signals across time, using the 1854 concepts as a distribution. Similar to the previous analysis with the MEG data, the BOLD activity from the fMRI recordings was correlated with the synthetic EEG activity. THINGS hemodynamic activation from V1, V2, V3 and the right fusiform face area was correlated with Concept2Brain output from simulated Oz and PO8 electrodes for each time point, across the 720 objects. The N170 face processing subset of the publicly available ERP CORE^40^ dataset was also utilized as an out-of-sample validation. Pictures of cars and faces were fed to the Concept2Brain model to generate a synthetic EEG dataset corresponding with ERP CORE. The correspondence between empirical and synthetic data was measured, focusing on amplitude differences at the right posterior electrode PO8, where the face-selective N170 component of the ERP is most pronounced.

Complementing this analysis, the ability of Concept2Brain to accurately predict EEG signals associated with facial expressions was tested using the KDEF dataset^53^. Here, 420 pictures from actors faces showing fearful and neutral expressions were fed into the model. The same actors appeared in both conditions, to control for differences in facial identity. In the resulting synthetic data set, we evaluated the expected differences between fearful and neutral conditions on components involved in facial expression processing (i.e., the N170 and the LPP components).

Fourth, we evaluated the generalization capabilities of the Concept2Brain model by employing written texts instead of pictures. To this end, 40 pleasant, 40 neutral, and 40 unpleasant short texts describing affective pictures were fed to the model for each participant. As a result, we obtained a synthetic EEG dataset of 10,560 trials showcasing electrophysiological differences.

Python scripts for Concept2Brain modeling, training and evaluation, along with MATLAB code for results visualization are available from: https://github.com/ASantosMayo/Concept2Brain. Trained Pytorch-based models and EEG dataset are accessible via https://osf.io/7dpgm. To facilitate the visualization and interpretability of the figures in the present work, a moving average filter with a window length of 40 ms was applied to the time series depicted in this report.

### The Concept2Brain cloud platform

The present project provides a new tool for generating open, flexible, and validated synthetic EEG datasets for the neuroscience community. We deployed our AI architecture in a cloud computing platform, allowing users to easily access the tool via the World Wide Web: https://concept2brain.brainbodybehavior.science. The model deployed in the platform was trained using EEG information from all subjects (88) and all the pictures (360). The web platform allows users to upload their own pictures or introduce written texts and select any number of synthetic participants at the front end (Supplementary Fig. 1.4 left). Note that the original participants cannot be reconstructed because of random shuffling and because of trial averaging, which obscures any fingerprint inherent in the raw EEG. This information is sent to the backend server where the Pytorch model, i.e., the Concept2Brain model, generates the synthetic ERP signal belonging to this input (Supplementary Fig. 1.4 middle). These data are saved as a MATLAB matrix format file, a commonly used format in neuroscience research. Finally, these data are made available for download and may be visualized in a user-friendly online data viewer consisting of an interactive 3D brain and timeseries plots of the 129 EEG channels across the trial. The platform allows the generation of a dataset containing 88 synthetic participants or only one participant.

The platform was developed using React^86^, a JavaScript framework. The backend server was set using the Django rest framework^87^, a python-based library used to provide the application programming interface (API) that handle user requests. Functions from the Pytorch library^81^ were utilized; the MNE library^88^ was used for web-based visualization of EEG topographic results.

### Reporting summary

Further information on research design is available in the Nature Portfolio Reporting Summary linked to this article.

## Supplementary information

Supplementary Information

Peer Review file

Reporting Summary

## Data Availability

The empirical EEG datasets used for training and evaluating the Concept2Brain model, along with the generated synthetic neurophysiological responses underlying the main figures, have been deposited in the OSF repository: https://osf.io/7dpgm.

## Code availability

The complete source code for the Concept2Brain model architecture, training pipelines, and evaluation scripts is publicly available in a GitHub repository: https://github.com/ASantosMayo/Concept2Brain.
