# Supplementary material for: Concept2Brain: an AI model for predicting neurophysiological responses to text and pictures
Source: Nat Commun. 2026 Jul 23;17:8961. doi: 10.1038/s41467-026-75653-x (PMC13503920; doi:10.1038/s41467-026-75653-x)
Supplement: Supplementary file 1 [file 41467_2026_75653_MOESM1_ESM.pdf]

Supplementary Information 1

The Concept2Brain model relies on a variational autoencoder (VAE) model [1,2], a deep learning architecture characterized by its capacity to compress high-dimensional information into a low-dimensional space. Specifically, this architecture exhibits a network structure whereby the following layers tend to reduce their number of neurons reaching the bottleneck layer. Then, the structure progressively expands, mirroring the input. It is trained using a non-linear process to compress the most relevant information of electroencephalography (EEG) input into an activity vector of 256 neurons at the bottleneck. In the present supplementary, we conduct three interpretability analyses to explore how this information is encoded in the low-dimensional space.

Isolating the effect of each latent dimension on the physiological recordings

The Concept2Brain's VAE model is trained using the Kullback-Leibler (KL) divergence as regularization method[3], to force the latent dimensions into a normal distribution. Here, we first examined how the predicted electrophysiological time series were mapped onto the latent space. For this analysis, we systematically manipulated the content of the latent space and forwarded it to the output layers to generate new EEG samples (129 sensors, 750 time points). Because the VAE follows a normal distribution, we used z-scores to explore its latent space. Specifically, we manipulated the input vector to contain all zeros except one

dimension, which was set to values of -5, 0, or 5 to examine the impact of each dimension on the simulated data. The figure below shows this exploration of the latent space by modulating the value (-5, 0 or 5) of the first three latent dimensions. Timeseries shown correspond to electrode Pz.

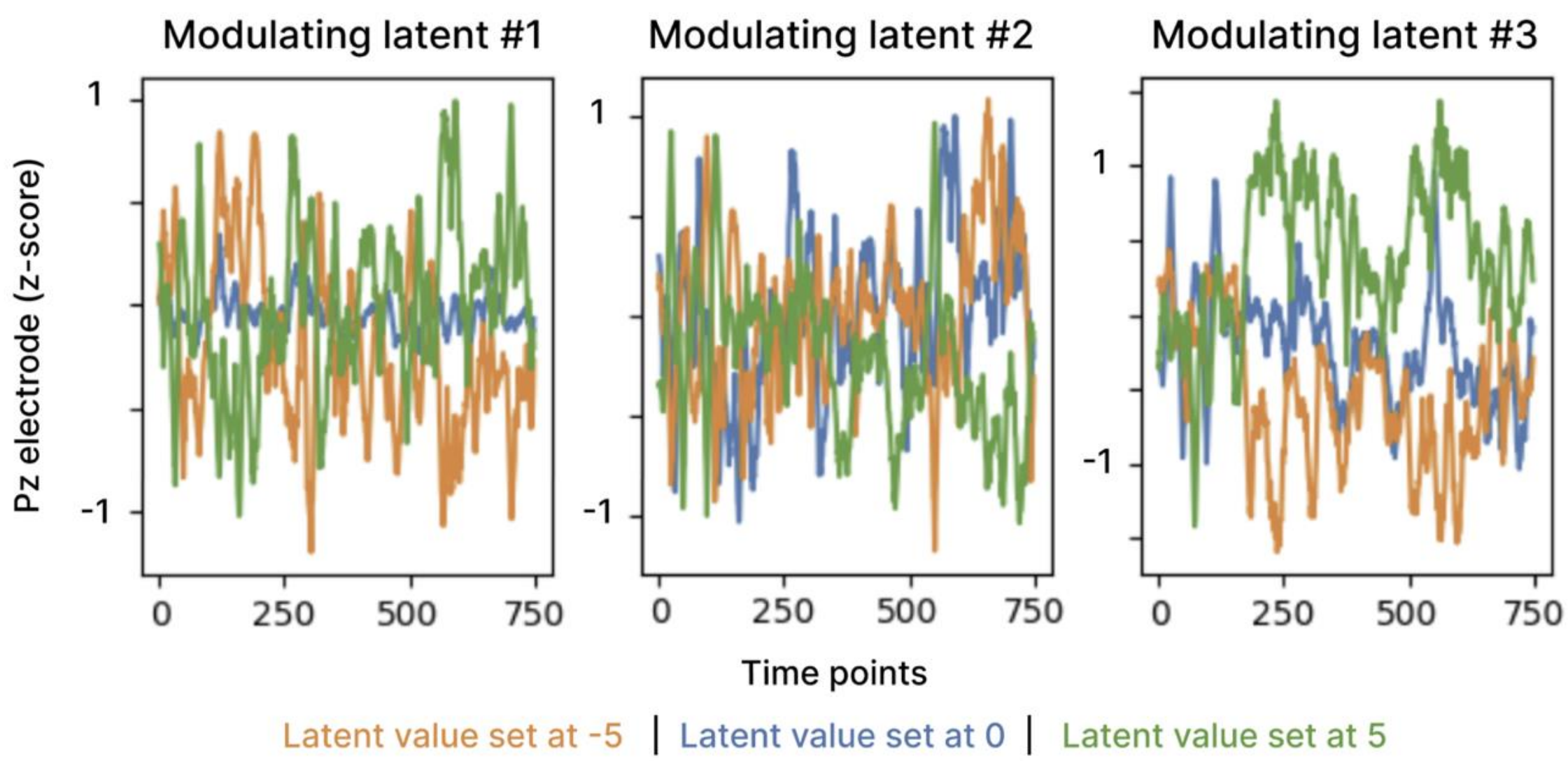

Figure SI1.1: **Electrophysiological signatures of latent dimension modulation.** Each one of the three subplots depicts the time series of electrode Pz when all dimensions, i.e. bottleneck neuron's values, are set to 0 except the three first neurons. By setting the value to -5 or 5, the dimension shows their extrems as it was previously confined to the Gaussian distribution thanks to the KL loss. Therefore, the subplots show how each latent dimension impacts on waveforms. The simulated time points (750) correspond with trial length of -0.1 to 1.4 s (frequency sample = 500 Hz).

In the example shown (Figure SI1.1), manipulating solely the third latent dimension creates amplitude modulations similar to those seen for empirical LPP component. However, the example shows that biological information such the LPP component is not only contained in one latent dimension.

Interpretability analysis on the latent space

Next, we conducted an analysis to characterize the interpretability of the information compressed within the VAE bottleneck. To this end, we estimated the 256 z values of the autoencoder for each one of the 360 pictures and each one of the 88 participants. These values were subsequently averaged across subjects, resulting in a representational matrix (360 images by 256 latent dimensions). A principal component analysis (PCA) was conducted over this information to visualize how the pictures are distributed in a 2-dimensional space (see Figure SI1.2).

As shown in the scatter plot, the reduced 2D space effectively clusters images according to their affective content: neutral images (gray) are concentrated in the upper-right quadrant, while pleasant (blue) and unpleasant (red) images occupy the upper-left and lower-left spaces, respectively. This organization indicates the preservation of representational information across the VAE bottleneck, maintaining the semantic and emotional structure of the original input.

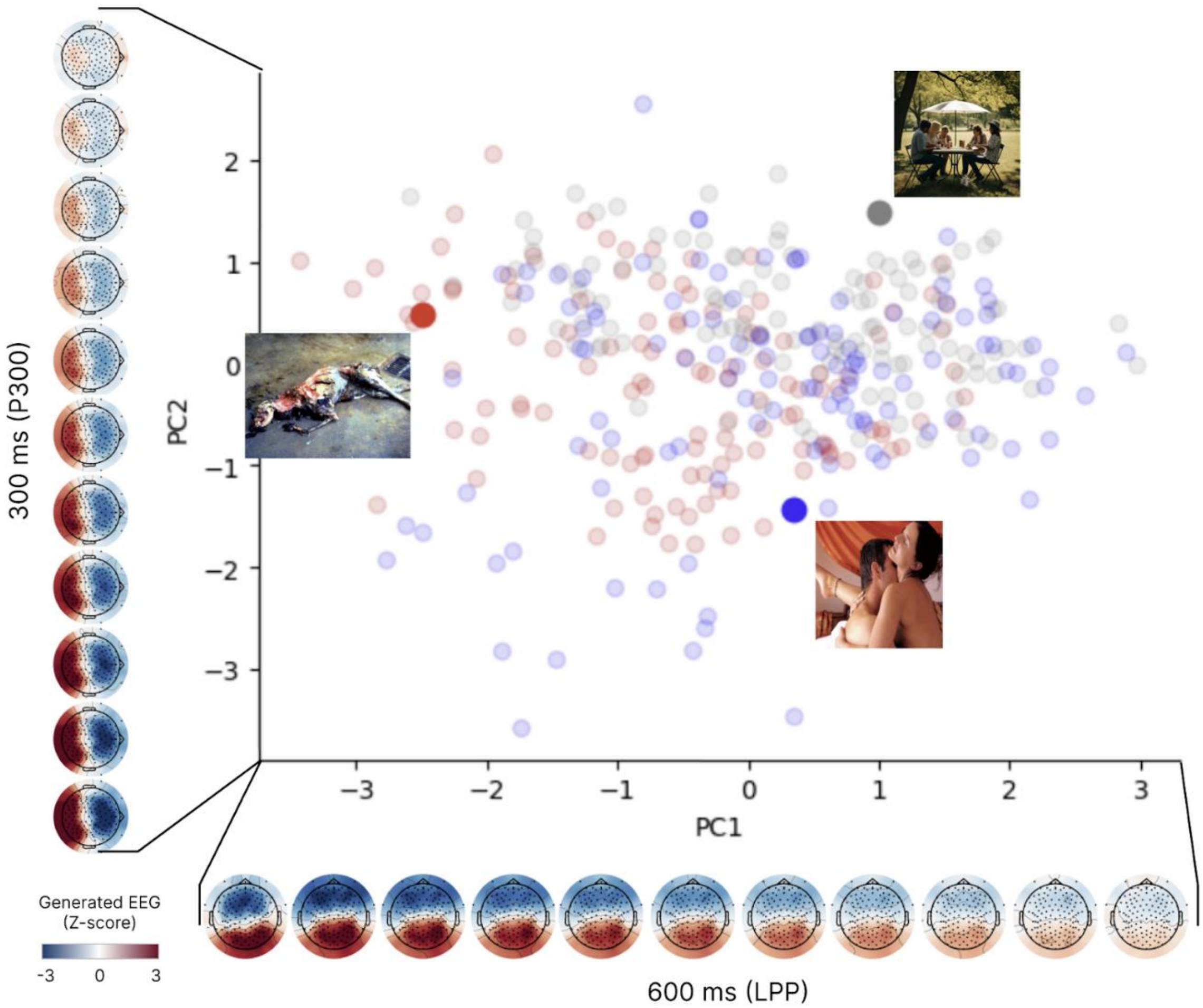

Figure SI1.2: **Latent space distribution of the emotional pictures using PCA.** The scatter plot shows the distribution of each one of the 360 pictures along two Principal Components (PC1-2). Blue, gray and red dots represents pleasant, neutral and unpleasant content of the pictures. Left: Topographies shows the scalp activity differences across the PC2 space. Specifically, the evoked amplitude increase takes place during aound 300 ms, matching the P300 component latency. Bottom: Topographies for the PC1 component shows similar scalp distributions than PC2. However, the PC1 component variance is found around 600 ms. It is congruent with the LPP component, an EEG modulation typically found in emotional processing.

Furthermore, we investigated the interpretability of the latent space by analyzing the mapping of the PC1 and PC2 components onto the picture space. To this end, we used a range of 11 points from PC1 (-3.5 to 3.5), leaving the PC2 set in 0, and calculating the reverse transformation of the PCA to estimate their 256 z values. Then, these values were used on

the VAE decoder to generate synthetic EEG data, sampling the PC1 space. The same procedure was repeated for PC2. Synthetic signals generated for each PC are shown along each axis (see Figure above). Resulting EEG signals showed a significant modulation in the evoked response amplitude at posterior sensors at around 600 ms for PC1 and around 300 ms for PC2. Results show the capability of the VAE architecture to successfully represent the two main sources of variability in the ERP responses to the 360 pictures, in an unsupervised fashion: the LPP and the P300 components.

Sparse Encoding of Electrophysiological Components

The prior analysis does not provide information about the individual contribution of each of the 256 latent dimensions to the generation of the simulated EEG signal. Therefore, an additional analysis investigated how each of the bottleneck neurons encodes relevant information of four major electrophysiological components (P100, P300, N170 and LPP). Specifically, we estimated Pearson's correlation coefficient between the EEG components of the synthetic data and the z-value of each of the VAE dimensions to quantify the relation between amplitude modulations in the simulated data and the content of the bottleneck. The figure below shows these correlation indices, with those likely differing from zero shown outside the gray area.

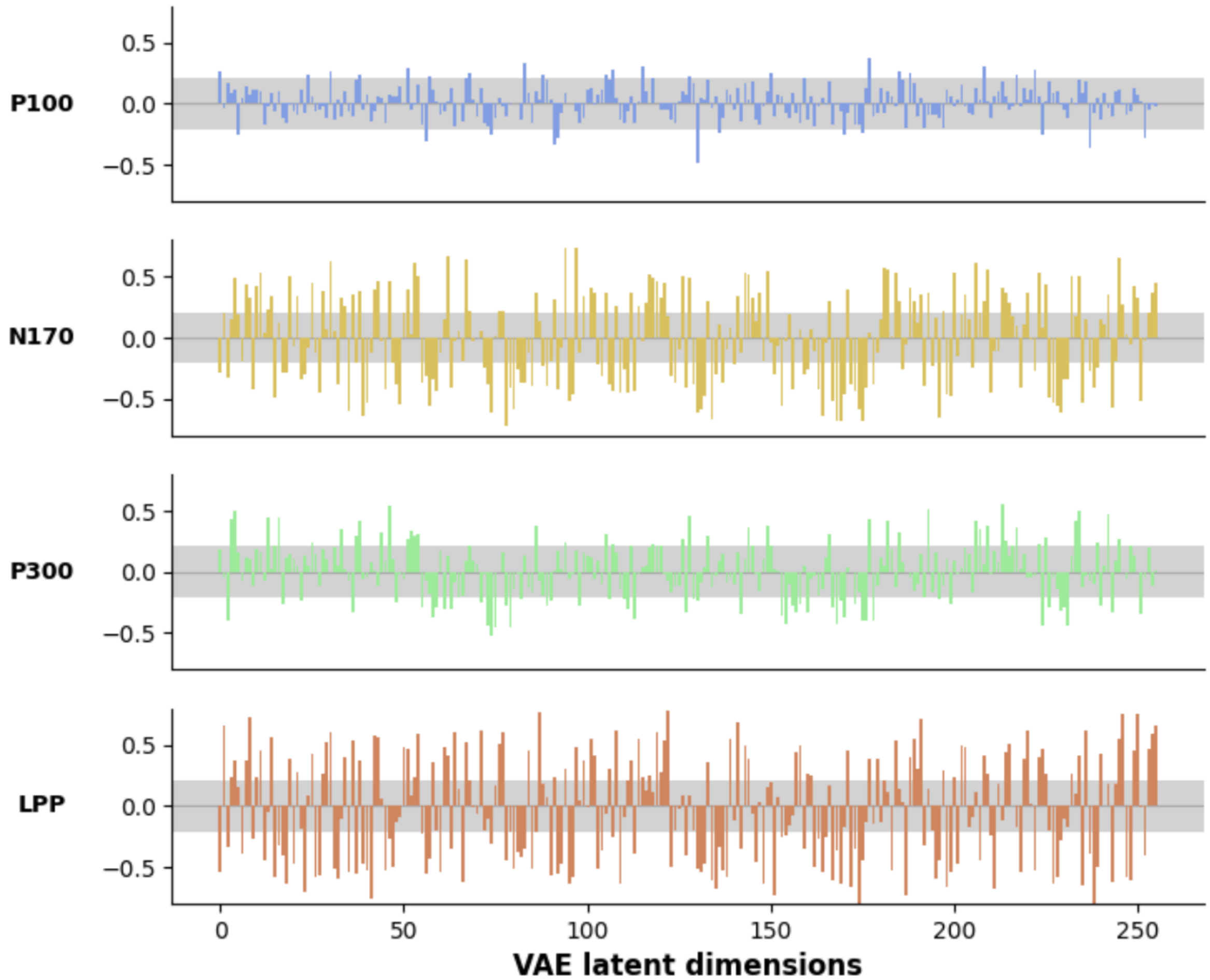

Figure SI1.3: **Pearson's correlation between latent dimensions and EEG components.** The bar plots show the contribution of each of the latent dimensions from the VAE's bottleneck to the amplitude of the main EEG components. Instead of showing the contribution of one or a few dimensions, the electrophysiological information seems to be encoded along the whole bottleneck. The gray area represents the 95% confidence interval (CI).

This analysis shows that the information required to reconstruct these signals is distributed across multiple dimensions. Consistent with the unsupervised nature of the VAE, the network stores the relevant information in a sparse fashion across the bottleneck neurons. It means that one neuron of the VAE does not map onto one EEG/ERP component or sensor, or a specific feature of the signal. Instead, the architecture stores the waveform information in form of activation patterns of the bottleneck neurons, and a given specific patterns

produces specific EEG signals. For example, in the case of the P100 component, its maximal correlation (Pearson's correlation -0.49) corresponds with the 131th latent dimension but the information is widely distributed across many additional latent dimensions, which are required for accurate P100 reconstruction. This is even more pronounced for the LPP component where a vast number of neurons encodes this information. The sparse but distributed representation of the electrophysiological information makes the VAE robust by forcing its decoder to integrate global activation patterns into bio-realistic EEG signals.

References

1. Kingma, D. P. & Welling, M. Auto-Encoding Variational Bayes. Preprint at https://doi.org/10.48550/ARXIV.1312.6114 (2013).
2. Bourlard, H. & Kabil, S. H. Autoencoders reloaded. *Biol Cybern* **116**, 389–406 (2022).
3. Mendonça, F., Mostafa, S. S., Morgado-Dias, F. & Ravelo-García, A. G. On the Use of Kullback–Leibler Divergence for Kernel Selection and Interpretation in Variational Autoencoders for Feature Creation. *Information* **14**, 571 (2023).
