# Supplementary material for: Concept2Brain: an AI model for predicting neurophysiological responses to text and pictures
Source: Nat Commun. 2026 Jul 23;17:8961. doi: 10.1038/s41467-026-75653-x (PMC13503920; doi:10.1038/s41467-026-75653-x)
Supplement: Supplementary file 2 [file 41467_2026_75653_MOESM2_ESM.pdf]

nature portfolio

Peer Review File

Concept2Brain: An AI model for predicting neurophysiological responses to text and pictures

Corresponding Author: Dr Alejandro Santos-Mayo

This file contains all reviewer reports in order by version, followed by all author rebuttals in order by version.

Version 0:

Reviewer comments:

Reviewer #1

(Remarks to the Author)
This is an interesting and potentially valuable paper. There is a growing need for models capable of generating high-quality synthetic EEG data, and the authors' attempt to simulate neural responses under varying semantic and emotional conditions is intriguing. The prospect of a model that can produce synthetic electrophysiological data in response to conceptual or perceptual stimuli is innovative and could have broad applications in computational neuroscience and brain-computer interface research.

However, I have a number of methodological and conceptual concerns that should be addressed to strengthen the paper.

1. Conceptual Clarity and Explainability

The proposed model currently operates effectively as a black box. It is unclear how the internal representations of the model relate to neurophysiological mechanisms or meaningful aspects of brain function. Without interpretability analyses or a link between latent dimensions and established neural processes, the model's scientific contribution remains limited. The authors could improve the paper by exploring what the learned representations correspond to (e.g., specific frequency bands, cortical regions, or ERP components). In its current form, the model demonstrates technical feasibility rather than explanatory insight.

2. Terminology

Throughout the manuscript, the term subject should be replaced with participant in keeping with current ethical and scientific conventions.

3. Performance Evaluation and Metrics

Performance is assessed primarily through correlations between grand-average ERPs derived from real and synthetic data. While this provides a basic indication of similarity, it offers only a partial view of the model's performance. A more comprehensive evaluation should include:

- Comparisons in the frequency domain (e.g., power spectral density, oscillatory components);

- Spatial/topographical similarity across electrodes;

- Temporal metrics such as peak latencies and amplitudes;

- Statistical comparisons of variance across trials and participants (e.g., using tests on distributions shown in the violin plots).

Together, such analyses would provide a fuller understanding of whether the synthetic data capture not just mean ERP shapes, but also realistic variability and higher-order EEG features.

4. Comparison with Existing Models

The manuscript would benefit from benchmarking against existing models of synthetic EEG generation, such as the Jansen–Rit neural mass model and its derivatives. These classical approaches explicitly model underlying neural dynamics, providing a useful point of comparison for assessing whether deep learning–based simulations add biological realism or only data-driven surface similarity. A deeper engagement with the background literature on synthetic EEG generation would contextualize the novelty and advantages of manuscript.

5. Nature of the Generated Signals

It is unclear, from reading the paper, whether the model generates full EEG signals or only event-related potentials (ERPs). The text alternates between references to “simulated EEG” and “ERP data,” which have distinct meanings. The authors should clarify whether ongoing (non-phase-locked) EEG activity is modelled, or whether the system only reproduces time-locked ERPs. If the latter, the title and abstract may currently overstate the scope of the model.

6. Dataset Description and Experimental Design

Important details about the empirical datasets are missing or underspecified. The methods should clearly describe:

- The number of trials per participant and any variations across participants;

- Whether image presentation order was randomized or counterbalanced;

- The precise trial timeline (e.g., stimulus duration, inter-trial interval);

- How emotional or semantic labels were defined—specifically, whether affective ratings were taken from the IAPS database or directly from participants.

7. Ethics and Participant Information

There is no mention of ethical approval, informed consent, or participant demographics (age range, gender distribution, inclusion/exclusion criteria). Such information is essential for studies involving human EEG data and should be explicitly reported.

8. Model Transparency and Ablation Analyses

The model description, while conceptually clear, lacks sufficient technical specificity. The number of filters, layer sizes, stride parameters, and output dimensions are missing or, in some cases, only briefly mentioned, which limits reproducibility. Moreover, it is unclear which architectural components are critical to performance. An ablation analysis (e.g., removing the conditioning layer or varying latent dimensionality) would help demonstrate which aspects of the architecture meaningfully contribute to model accuracy and generalization.

9. Semantic and Emotional Labeling

The paper should clarify how “semantic” and “emotional” content were quantified. Were labels derived automatically from CLIP embeddings, or based on pre-existing IAPS ratings? If the latter, emotional valence and arousal can differ substantially from participant-reported experience. Explicitly defining these constructs and their sources is necessary for scientific transparency.

10. EEG Preprocessing and Referencing

Key preprocessing details are missing or ambiguous. There is no information about referencing schemes after interpolation (e.g., average reference or Cz reference), baseline correction procedures, or criteria for trial exclusion. These choices have a significant impact on the resulting ERPs and on how well synthetic data can be compared with real EEG.

11. Cross-Validation and Generalization

There is no clear cross-validation or hold-out testing strategy. A clear validation scheme (e.g., leave-one-participant-out or leave-one-stimulus-out) would provide stronger evidence of the model’s robustness.

12. Model Complexity and Risk of Overfitting

Given the relatively small dataset (~13,600 trials) and the apparent size of the neural architecture, there is a significant risk of overfitting. The paper does not mention the use of regularization methods such as dropout, early stopping, or weight decay. Including these details—and ideally reporting training and validation loss curves—would help assess whether the model truly generalizes beyond the training data.

Summary

In summary, the Concept2Brain model is an ambitious and promising step toward data-driven simulation of brain activity.

However, the current manuscript lacks crucial methodological detail, interpretability, and rigorous validation. Clarifying the exact scope of the model (EEG vs. ERP), providing fuller dataset and participant information, expanding evaluation metrics, implementing clear cross-validation, and situating the work within the broader literature on synthetic EEG generation would substantially strengthen the contribution.

(Remarks on code availability)
The code is available on github and, when downloaded, appears to function well.

Reviewer #2

(Remarks to the Author)
This paper purports to provide "a computational architecture for converting information contained in a text or image into synthetic ERP waveforms." The model and its evaluation do not, however, live up to these claims.

The model is trained on a total of 360 images. This is extremely insufficient to claim that the model is suitable as a general EEG encoder. The amount of data also appears insufficient to learn to approximate individual subjects given that an individual actually only has data from 120 or 240 images.

The authors do not show training curves for the model or report the train or test MSE, making it hard to evaluate the model's performance directly. Furthermore, it is not clear how the train/test split was done. It seems that it is *not* the case that certain images were held out entirely, but rather the data was arranged as subject-image combinations and these were randomly split. This represents only the most minimal test of generalization, and even the results on this test set were not shown. Validation data is not discussed at all. The ability of the model to replicate specific individuals is also not analyzed.

As far as I can tell, the results in Fig2A-D are evaluated primarily on training data (it seems that all images were used for these analyses, meaning that 80% of the data used is training data). The performance of the model on training data is not relevant or appropriate to report; it is not indicative of the model's general abilities (and even when evaluating on training data the model still doesn't seem to generally correlate well with the real EEG, though this is not fully reported). Fig 2E appears to actually use images not trained on, but the actual fit of the model to EEG data is not reported and the correlation to the arousal metric is quite weak. Fig 4F-G appears to use non-training images, but again does not report fit to EEG data, but rather demonstrates a simple qualitative finding from the literature. Therefore these results do not help support the idea that this model has learned a general ability to predict EEG data in response to any image.

The authors refer to the text-based simulation as "replicating an entire experiment", but it is not a replication if the experiment was never done, and indeed none of the results are compared to any text-based experimental results. Rather, the model outputs are qualitatively compared to image-based experiments. Given this and the nature of CLIP, it seems that this model would not do well in predicting how EEG signals differ when text vs images are used, and therefore it is not a good encoder model for EEG.

Fig 5 shows that affect can be decoded somewhat from the simulated EEG signals. It is unclear what this indicates about the model as these results are not directly compared to real data. The absolute decoding performance is not indicative of the quality of the EEG model specifically because it is probably the case that affect could be just as well if not better decoded from CLIP output alone.

In total, the model, when not evaluated on training data, shows some qualitative similarity to EEG signals specifically around affect. This does not constitute a "an AI model for predicting
neurophysiological responses to text and pictures" in any general sense. The authors claim that through the model "differences can be examined at the level of individual prompts and individual participants, without the need of large datasets" and that "in clinical studies, this model may
be used to generate typical brain responses for initial comparisons with a patient group". There is no evidence provided to support these claims and in fact the latter would be incredibly inappropriate and potentially damaging.

(Remarks on code availability)

Reviewer #3

(Remarks to the Author)
This paper presents a methods to generate visual ERPs from images (and other content) using a variational autoencoder and a connection to OpenAI CLIP platform. Overall I think it's a useful contribution, but I have some comments and suggestions.

1. Presentation: My main critique of this paper is that the presentation of the text is quite suboptimal. At the moment, if feels the flow is rather disorganised, forcing the reader to go back and forth to understand things:
-> The Results assume the reader knows things that are only explained later. As an example, please provide *conceptual* details on the conditioned Variational Autoencoder, the electrophysiological latent space, and the cross-domain neural network, as well as anything that is needed to get a basic understanding of the Results. The purpose and meaning of these have to be clear to a non-technical reader.

-> Datasets 1 and 2 are not explained until much later on, which doesn't help.
-> In Fig2, the authors first talk about panel A, then E, then BCD. And from then they jump to Fig6. It would be a good exercise to try to describe the figures sequentially, I think this would improve the flow substantially.
-> Some results (fig5) are presented in Discussion instead of Results which it's quite unorthodox and also a bit confusing.
These are just examples: in general please do a careful read of the whole manuscript trying to

2. Subject- vs. group-level results: I think a very important panel for the validation of this paper is 2D because it's most quantitative result showing that this works. If every dot is a time point / subject, it would be very useful to use colours to indicate time, and indicate the correlation in the Figure. Also, this aggregates all subjects in a single measurement. What about correlations for each subject? It would be useful to show a distribution of those, perhaps across all time points. I understnd the point of this is group-level results, but in general, I'm missing subject-specific results, which are also important scientifically.

3. Validation: Overall, the authors showed a set of cases where the model produces responses that make sense in one way or another. I would guess these were not the first and only tests that the authors have tried, and that other things didn't work - which is absolutely normal and to be expected. Now my question is: how often does it work? When it didn't work? It would be very useful to know the limits of the model as well. In this line, for example, what would it happen in the result shown in Fig2D if the model is trained on a subset of the subjects and tested on unseen subjects? (I understand the scatterplot is from subjects that were used in both train and test). Of course the latter results are shown for data that was not used in training, but they are rather qualitive and cherry-picking on very specific characteristics of the ERP. Would an equivalent of Fig 2D work for unseen data?
4. Beyond the averages: Do the authors have any comment about aspects that go above and beyond the ERP average? There is a lot of information at the trial basis, and other simulation methods, like Genephys (Vidaurre, 2024 *) work at the level of trials instead of averages. What would be the pros and cons of each approach with respect to each other? In general, disentangling the different contributions to the effect requires having trial-basis signals (Vidaurre, Cichy, Woolrich; 2021 **), which I would understand as a limitation of Concept2Brain. Of course, I understand capturing between-trial variability is beyond scope, so I'm only asking to discuss this.

* https://elifesciences.org/articles/87729
** https://academic.oup.com/cercor/article-abstract/31/12/5664/6324865

(Apologies here for citing my own work, it's just the things that come to mind)

5. Section "Electrophysiological response based on emotional text": I don't quite get the scientific use of this. The proposed model is about generating responses to visual stimulation. The fact that CLIP can draw links between text and images seems beyond the merits and capacities of the proposed model, other than a practical convenience if the user do not have images at hand; but, again, other ready-to-use tools exist for that. Please clarify.

6. Section "ERP generation: Web implementation": I find the section slightly confusing. As I understand, the web implementation differs in the interface (web vs non-web), but the engine is essentially the same. Why does it then need a separate demonstration with another set of stimuli, resulting ERPs, etc? Overall, Fig4 mixes another "scientific demo" (alligator, desk and beach) with showing that it also runs on web - but you don't need another set of stimuli for that. If this new set of stimuli does bring something new to the story, or even if it just offers further empirical validation, but there's nothing specific about being run through the web, I would put it with the other demonstration and just say that Concept2Brain can run on the web.

7. Example on conceptual content in Fig 4D. Following on the comment above, the fact that the pipeline can generate ERPs from complex sentences isn't surprising given that CLIP can basically link anything to anything. So the authors' approach will always generate something anyway. The more interesting question is why it can generate an effect between this negatively arousal sentence and another that is more neutral or even positive: To which images in the training data set on which Concept2Brain was trained this is being linked to? (This is also confusing to be mixed with the web feature by the way.)

Minor / detail comments:

- "Instead of decoding brain activity to classify or reconstruct a given stimulus, event, or condition, this approach intends to encode the stimulus to generate bio-realistic brain activity."

It would be useful to make the distinction of models to predict brain activity using features from the stimuli from actual simulation methods to actual simulate brain data given a set of hyperparameters.

- Gifford AT, Bersch D, Roig G, Cichy RM. The Neural Encoding Simulation Toolkit.

I couldn't find this reference

- "If information is systematically shared between real world concepts expressed in media and text (conceptual latent space in Figure 1.2) and their representation in the human brain"

There's quite a leap from the previous text in the paragraph to this statement. This is the first occurrence of "media and text". At this stage it is unclear what media and text -and in general, language- have to do with ERPS/encoding/decoding methods in Neuroscience. Please clarify: what is the rationale, assumptions, motivation, etc of such link?

- LPP: define in the first use (it says Late Positive Potential component a few lines below it's used for the first time). Both in caption and main text

- Fig 2BC, add legend for colours

- Caption 2. "normative arousal ratings collected in a standardization sample". Some more explanation is needed to understand what this refers to.

- Fig 2E: The explanation seems insufficient, both in main text and caption

- "showed a statistically significant correlation of r = 0.23". Be clear between what and what.

(Remarks on code availability)

Reviewer #4

(Remarks to the Author)
Santos-Mayo et al propose a new model to predict EEG responses to images.

The authors introduce and train a 2-stage model to learn (1) latent EEG responses and (2) condition them with a visual or textual prompt paired with a pretrained image embedding model. The result is the possibility to predict the event related potential in response to an image, or its corresponding caption.

Overall, the study tackles a timely objective. However, the data, methods, results and contributions remain modest, and suggest that this work may be more suitable for a specialized journal or conference.

Major:

Data

This study is evaluated on a modest image dataset:
- 45 subjects watching 120 pictures, and
- 43 subjects watching 240 images:

This is substantially lower than previous studies:
- Teichmann et al and Hebart (MEG): 10 subjects 22k images
- Grootswagers et al (EEG 2022): 50 subjects watching 22k images
- Gifford et al (EEG 2022): 10 subjects watching 16k images.

This small dataset means that the model can only learn the brain responses to a small diversity of image. Consequently, it would, even in principle, be difficult for the model to generalize to a large set of naturalistic images / prompts.

Method:

The train/test method may be partly invalid: The authors indicate that the training set is 80% of the EEG data (10K trials).

However, the (120 or 240) images are repeated across participants.

To ensure that the model does not learn the association between EEG and images "by heart", it is necessary to perform these experiments with out-of-image predictions (as in Allen et al 2022).

For example, if we assume that there is an EEG component shared across participants (e.g. the famous N170 specific to faces), then the model could learn that for images A, B, C, it should predict a N170. When evaluated on (held out) EEG responses to the same A, B and C images, it would then be above chance.

Specifically, the authors need to do a group splitting based on the image at line:
https://github.com/ASantosMayo/Concept2Brain/blob/main/Concept2Brain_training_script.py#L178

Results

First, there are several perfectible statements : “For example, the simulated ERP at the mid-occipital Oz electrode shows deflections consistent with early visuo-cortical responses to stimulus onset, whereas the simulated data at the mid-parietal

Pz electrode contains the late positive potential component, modulated by emotional content. Also consistent with empirical data, the ERP waveforms show pronounced differences across participants."

-> These elements are not thoroughly evaluated with statistical methods.

Second, the main validations of the models stand on two main results:
1. LPP components correlate with the normative arousal ratings for each picture.
2. A modulation of the prediction depending on the presence of a face.

While interesting, these two elements are quite limited compared to the scope of building a predictive model of EEG responses to natural images.

Contributions:

The authors introduce a new encoding model, but do not compare it to baseline models e.g. linear TRF, or deep encoder etc (e.g. Gifford et al 2022, Benchetrit et al arxiv 2025, O Chehab, Defossez et al 2022).

Minor:

- The introduction could be substantially shortened: e.g. by perhaps limiting the historical perspective of the invention of EEG and its various use cases, and the explanation of what an evoked response is.

- The architecture design are not motivated: the authors should provide ablation experiments to evaluate the utility of their choices (number of layers, multi stage fitting etc)

- "However, despite their potential, encoding models for EEG remain relatively under-explored, with many questions regarding their ability to accurately replicate the complexity of real brain activity."
-> It would be great to detail further these remaining questions to clarify the contributions.

- Pearson's correlation $r = 0.640$, p-value < 0.01
-> Given the strength of this correlation, and following Fig2E caption, I understand that this statistics is performed on the grand ERP average across subjects. However, a valid statistics would require being tested across the 88 subjects, since the other dimensions (channels, times, etc) are not independent from one another

(Remarks on code availability)

Version 1:

Reviewer comments:

Reviewer #1

(Remarks to the Author)
I am happy that the reviewers have addressed all my comments. I recommend acceptance.

(Remarks on code availability)

Reviewer #2

(Remarks to the Author)
The authors have done substantial additional work to properly train and test the performance of the model. This is useful for readers to understand its strengths and limitations. I am still concerned that some results are over-stated or not correctly contextualized and that this may misguide readers. Specifically, the new out of distribution tests done on the THINGS and ERP-Core datasets are important tests, but the description by the authors does not acknowledge the substantially lower correlation between the model and data compared to the within distribution test results. Rather, these results are described as "strong". The authors should accurately acknowledge that out of distribution performance is weaker, and that users of Concept2Brain should proceed carefully as a result. Relatedly, the paper should include a much clearer disclaimer that the usage of text as input has not been verified in anyway, and so this use case is possible with this model, but not validated.

Given that the authors are now correctly cross-validating their model by using held out images, I am slightly confused about which version of the model is used for the web interface. Specifically, for the results in Figures 1 and 2, 10 different versions of the model were trained (one for each fold). Which version of the model is run when using the interface?

(Remarks on code availability)

Reviewer #3

(Remarks to the Author)
The authors have responded to my questions satisfactorily for the most part.
I'd like to follow up on point number 4, though.

"The newly added analysis of oscillatory content (Figure 3D) demonstrates that some information from the individual trials, such as aspects of induced oscillations, is contained in the synthetic data".
A time-frequency analysis of the data is totally based on averages, so there's no account of trial-specific variability in there - unless I'm missing something?

"In the revised manuscript, we acknowledge this fact and emphasize that the lack of single-trial variability in response to the same stimulus represents a problem for many advanced single-trial analyses".
Apologies if I have missed something but I can't find any of this in the new text. Is it possible that it slipped?

(Remarks on code availability)

Open Access This Peer Review File is licensed under a Creative Commons Attribution 4.0 International License, which permits use, sharing, adaptation, distribution and reproduction in any medium or format, as long as you give appropriate credit to the original author(s) and the source, provide a link to the Creative Commons license, and indicate if changes were made.
In cases where reviewers are anonymous, credit should be given to 'Anonymous Referee' and the source.
The images or other third party material in this Peer Review File are included in the article's Creative Commons license, unless indicated otherwise in a credit line to the material. If material is not included in the article's Creative Commons license and your intended use is not permitted by statutory regulation or exceeds the permitted use, you will need to obtain permission directly from the copyright holder.
To view a copy of this license, visit https://creativecommons.org/licenses/by/4.0/

UF UNIVERSITY of FLORIDA

College of Liberal Arts and Sciences
Department of Psychology
Laboratory for Brain, Body, Behavior

Gainesville, FL 32611
Phone: 352-273-0843
3/18/2026

REVIEWER COMMENTS

Reviewer #1 (Remarks to the Author):

This is an interesting and potentially valuable paper. There is a growing need for models capable of generating high-quality synthetic EEG data, and the authors' attempt to simulate neural responses under varying semantic and emotional conditions is intriguing. The prospect of a model that can produce synthetic electrophysiological data in response to conceptual or perceptual stimuli is innovative and could have broad applications in computational neuroscience and brain-computer interface research.

However, I have a number of methodological and conceptual concerns that should be addressed to strengthen the paper.

1. Conceptual Clarity and Explainability

The proposed model currently operates effectively as a black box. It is unclear how the internal representations of the model relate to neurophysiological mechanisms or meaningful aspects of brain function. Without interpretability analyses or a link between latent dimensions and established neural processes, the model's scientific contribution remains limited. The authors could improve the paper by exploring what the learned representations correspond to (e.g., specific frequency bands, cortical regions, or ERP components). In its current form, the model demonstrates technical feasibility rather than explanatory insight.

Response: We agree with this sentiment and have attempted to add as much information regarding the internal workings and mechanistic interpretations to the revision as possible. Given space limitations however, a Supporting Information 1 appendix has been added in the new version. Several aspects have been added: During the creation of the Concept2Brain model we investigated how the conditioned VAE architecture translates into the simulated electrophysiological information. This is now described in greater detail in the revised manuscript. We first trained the autoencoder architecture using the Kullback-Leibler loss as regularization method (Mendonça et al., 2023), to force the latent dimensions into a

normal distribution. Here, we examined how the predicted electrophysiological time series were mapped onto the latent space. For this analysis, we systematically manipulated the content of the latent space and forwarded it to the output layers to generate new EEG samples (129 sensors, 750 time points). Because the VAE followed a normal distribution, we used z-scores to explore its latent space. Specifically, we manipulated the input vector to contain all zeros except one dimension, which was set to values of -5, 0, or 5 to examine the impact of each dimension on the simulated data. The figure below shows this exploration of the latent space by modulating the value (-5, 0 or 5) of the first three latent dimensions. Timeseries shown correspond to electrode Pz. In this example, manipulating solely the third latent dimension creates amplitude modulations similar to those seen for empirical LPP component.

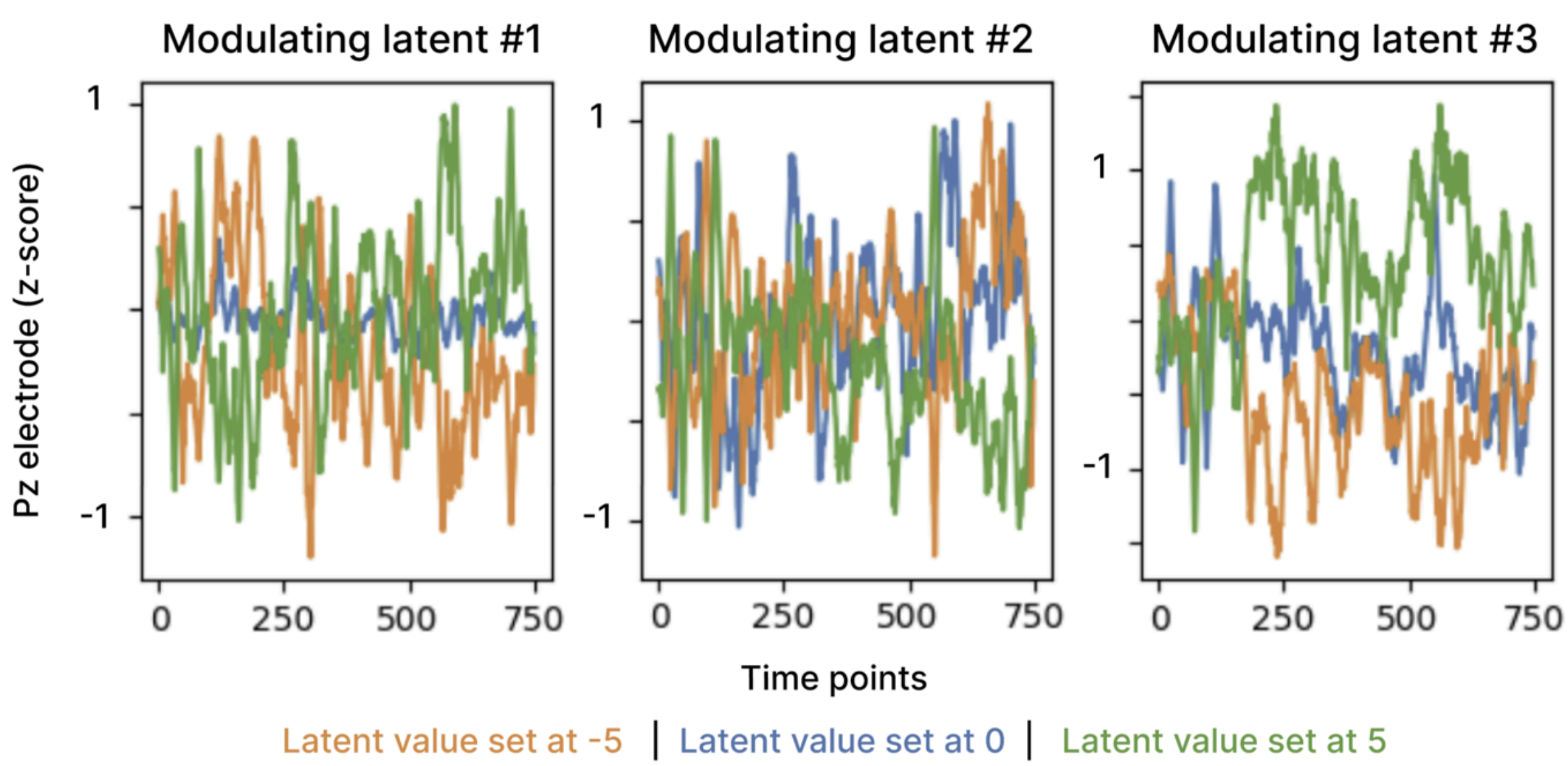

Next, we conducted an analysis to characterize the interpretability of the information compressed within the VAE bottleneck. To this end, we estimated the 256 z values of the autoencoder for each one of the 360 pictures and each one of the 88 participants. These values were subsequently averaged across participants, resulting in a representational matrix (360 images by 256 latent dimensions). A principal component analysis (PCA) was conducted over this information to visualize how the pictures are distributed in a 2-dimensional space (see Figure below).

As shown in the scatter plot, the reduced 2D space effectively clusters images according to their affective content: neutral images (gray) are concentrated in the upper-right quadrant, while pleasant (blue) and unpleasant (red) images occupy the upper-left and lower-left spaces, respectively. This organization indicates the preservation of representational

information across the VAE bottleneck, maintaining the semantic and emotional structure of the original input.

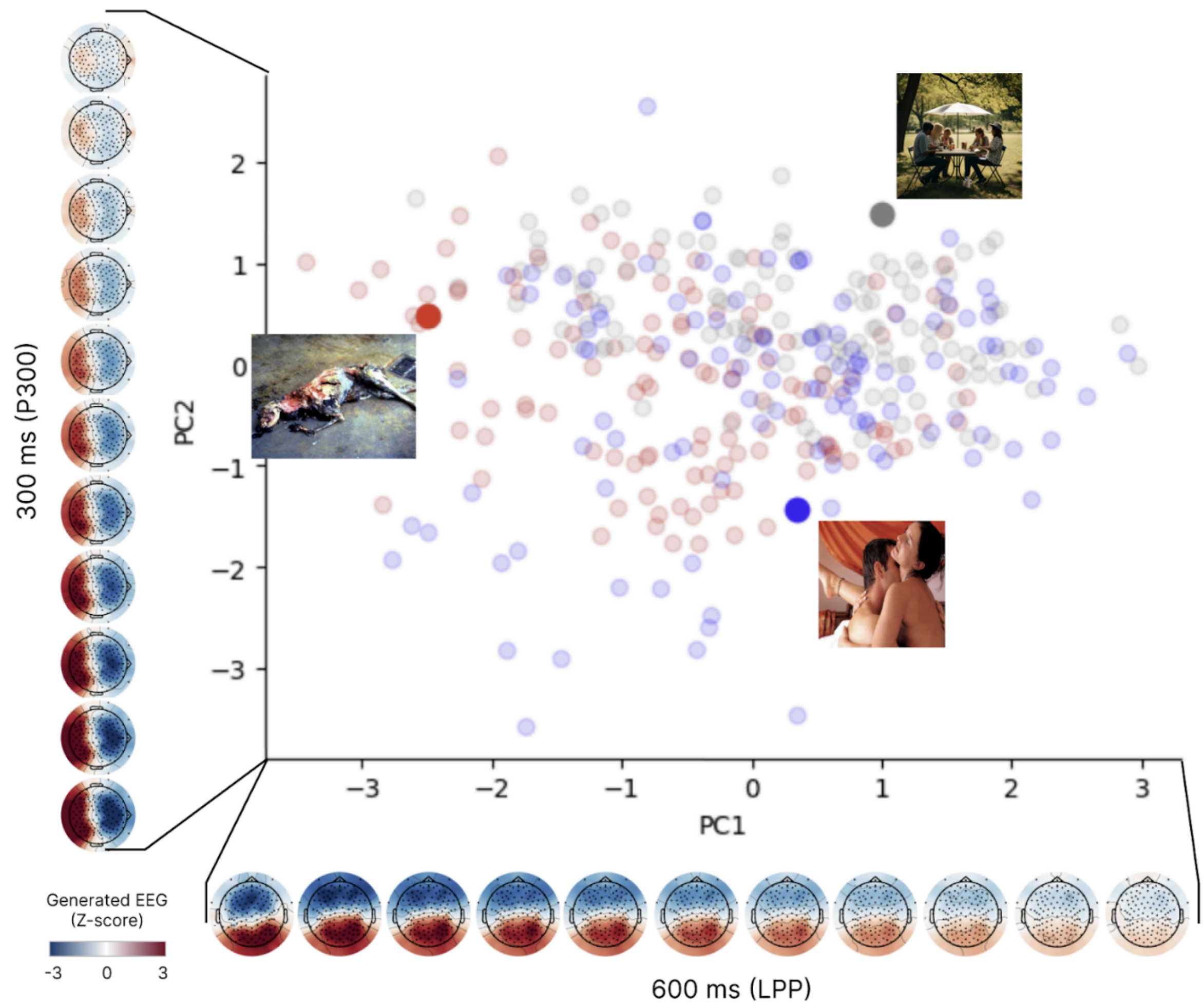

Furthermore, we investigated the interpretability of the latent space by analyzing the mapping of the PC1 and PC2 components onto the picture space. To this end, we used a range of 11 points from PC1 (-3.5 to 3.5), leaving the PC2 set in 0, and calculating the reverse transformation of the PCA to estimate their 256 z values. Then, these values were used on the VAE decoder to generate synthetic EEG data, sampling the PC1 space. The same procedure was repeated for PC2. Synthetic signals generated for each PC are shown along each axis (see Figure above). Resulting EEG signals showed a significant modulation in the evoked response amplitude at posterior sensors at around 600 ms for PC1 and around 300 ms for PC2. Results show the capability of the VAE architecture to successfully

represent the two main sources of variability in the ERP responses to the 360 pictures, in an unsupervised fashion: the LPP and the P300 components.

However, the previous analysis does not provide information about the individual contribution of each one of the 256 latent dimensions to the generation of the EEG signal. Therefore, our following analysis investigated how each one of the bottleneck neurons encodes relevant information of four widely known electrophysiological components (P100, P300, N170 and LPP). Specifically, we estimated the Pearson's correlation coefficient between the EEG components of the synthetic data and the z-value of each one of the VAE dimensions. As a result, we can study the actual contribution of each one of the dimensions to generate a higher or lower amplitude in such components. The figure below shows correlation indices, being any point above or above the gray square statistically significant (α=0.05).

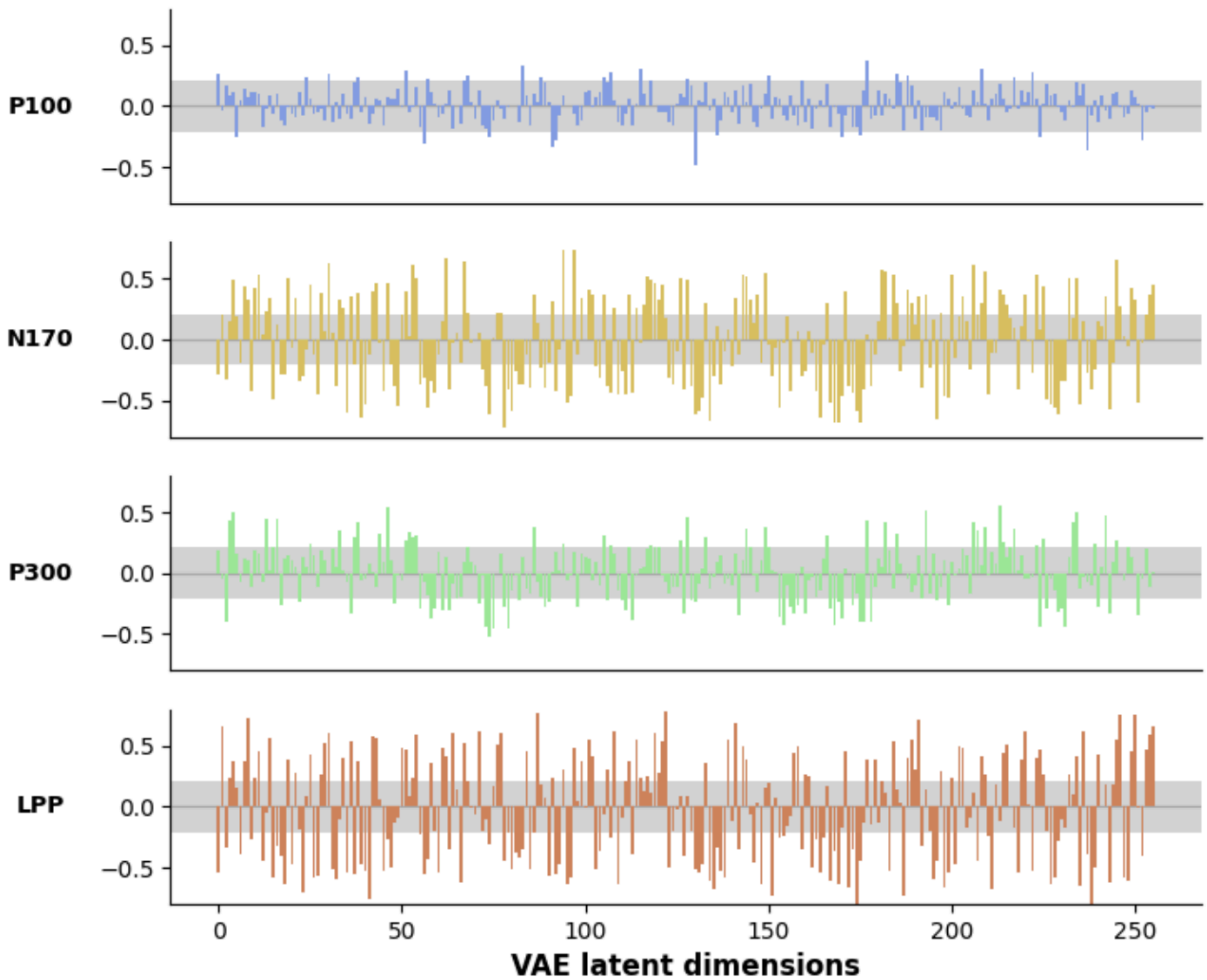

This analysis shows that the information required to reconstruct these signals is distributed across multiple dimensions. Consistent with the unsupervised nature of the VAE, the network stores the relevant information in a sparse fashion across the bottleneck neurons. It means that one neuron of the VAE does not map onto one EEG/ERP component or sensor, or a specific feature of the signal. Instead, the architecture stores the waveform information in form of activation patterns of the bottleneck neurons, and a given specific patterns produces specific EEG signals. For example, in the case of the P100 component, its maximal correlation (Pearson's correlation -0.49) corresponds with the 131th latent dimension but the information is widely distributed across many additional latent dimensions, which are required for accurate P100 reconstruction. This is even more pronounced for the LPP component where a vast number of neurons encodes this information. The sparse but distributed representation of the electrophysiological information makes the VAE robust by forcing its decoder to integrate global activation patterns into bio-realistic EEG signals.

The results of the interpretability analyses above are now provided in Supplementary Information 1.

2. Terminology

Throughout the manuscript, the term subject should be replaced with participant in keeping with current ethical and scientific conventions.

Thank you for the suggestion; now the manuscript and figures have been updated with this term.

3. Performance Evaluation and Metrics

Performance is assessed primarily through correlations between grand-average ERPs derived from real and synthetic data. While this provides a basic indication of similarity, it offers only a partial view of the model's performance. A more comprehensive evaluation should include:

- Comparisons in the frequency domain (e.g., power spectral density, oscillatory components);

This is a great suggestion. To examine the reproducibility of empirical oscillatory phenomena through Concept2Brain, we conducted a time-frequency analysis of a subset of simulated and empirical data with 40 pictures taken from the pleasant condition from the first 45 subjects corresponding to the first dataset.

The new Figure 3 C shows the results for both the empirical EEG and synthetic EEG in response to the same pictures, for electrode site Oz. Several key features of oscillatory responses to complex scenes are visible in both representations, as is now detailed in the revised manuscript. For example, the expected alpha power reduction in response to picture onset is clearly visible in empirical and synthetic data. The plots also illustrate differences in overall scale between empirical and synthetic data, with the Concept2Brain model providing a version of the signal that possesses high signal-to-noise though regularization across exemplars and participants.

The analysis suggests that the cVAE model and the convolutional layers are capable of capturing characteristic time-frequency features of data and then introduce this subtle frequency-related features into the generated output.

- Spatial/topographical similarity across electrodes;

In response to this comment, we have added a cross-validation analysis (see also below for more details) in which we directly estimated the spatial similarity between real and synthetic participants across electrodes. This analysis is now shown in the new Figure 2 F.

- Temporal metrics such as peak latencies and amplitudes;

We provide correlational analyses across time points, which capture the latency and time-varying amplitude modulations as requested. Because latency estimation of ERPs varies with signal-to-noise and requires targeted methods such as jackknifing that require extensive description, we did not add dedicated analyses of the latency of ERP components. We are however happy to include those analyses if the reviewer and editor deem them helpful.

- Statistical comparisons of variance across trials and participants (e.g., using tests on distributions shown in the violin plots).

In the new version of the manuscript, we have added additional illustrations of variability across trials using Violin plots. One of the particularities of the Concept2Brain model is its capacity to extract the stimulus-specific signal from noise and regularize the output though using observations across exemplars and participants. Therefore, all participants exhibit similar patterns. For example, differences between cars and faces are seen for all the 88 synthetic participants while retaining their idiosyncratic waveforms across conditions. This is now better illustrated and discussed in the revised version.

Together, such analyses would provide a fuller understanding of whether the synthetic data capture not just mean ERP shapes, but also realistic variability and higher-order EEG features.

Thank you again, we believe that the additional analyses have strengthened the manuscript greatly.

4. Comparison with Existing Models

The manuscript would benefit from benchmarking against existing models of synthetic EEG generation, such as the Jansen–Rit neural mass model and its derivatives. These classical approaches explicitly model underlying neural dynamics, providing a useful point of comparison for assessing whether deep learning–based simulations add biological realism or only data-driven surface similarity. A deeper engagement with the background literature on synthetic EEG generation would contextualize the novelty and advantages of manuscript.

We agree that neural encoder models such as the Jansen-Rit mass model or spiking neural networks will generate bio-plausible electrophysiological recordings of the brain. Some of these models would allow users to simulate cortical surface activity and compute a forward model to the EEG sensors. We are now discussing in greater detail that there two aspects in which the Concept2Brain architecture adds to these available models: First, it creates a direct link between semantic content and electrophysiological responses. This is challenging for neural mass or spiking models that are typically not suited to accept complex semantic input. Second, models such as Jansen-Rit, spiking networks (Yamazaki et al., 2022) or the human neurocortical neurosolver (HNN; (Neymotin et al., 2020) excel in their ability to model neurophysiological processes such as excitatory-inhibitory balances and biophysical

properties of the brain and surrounding tissue. The strength of Concept2Brain is to focus on the replicability of the EEG signal without making assumptions regarding under the underlying physiology and physics.

These important points have been added to the current version of the manuscript.

5. Nature of the Generated Signals

It is unclear, from reading the paper, whether the model generates full EEG signals or only event-related potentials (ERPs). The text alternates between references to “simulated EEG” and “ERP data,” which have distinct meanings. The authors should clarify whether ongoing (non-phase-locked) EEG activity is modelled, or whether the system only reproduces time-locked ERPs. If the latter, the title and abstract may currently overstate the scope of the model.

We thank the reviewer for raising this important point. In the revised version, we now explain that the Concept2Brain model was trained on single-trial segments. Therefore, it reproduces single-trial output, which in an empirical study would be referred to as an EEG segment, not an ERP. However, the main strength of the autoencoder architecture is to capture the signal that is specific to a given input, resulting in outputs with an extremely high signal-to-noise ratio signal. As a result, the Concept2Brain output may at times resemble participant-level ERP signals, often showing typical ERP components. Nonetheless, this output still corresponds to one trial for one participant. An additional feature of the Concept2Brain architecture is its consistency, where, in a given participant, the same input will result in the same output; in other words, the output does not vary across repeated trials but varies across participants. Again, these aspects are now detailed in the revised version of the manuscript.

6. Dataset Description and Experimental Design

Important details about the empirical datasets are missing or underspecified. The methods should clearly describe:

- The number of trials per participant and any variations across participants;

- Whether image presentation order was randomized or counterbalanced.

- The precise trial timeline (e.g., stimulus duration, inter-trial interval);

- How emotional or semantic labels were defined—specifically, whether affective ratings were taken from the IAPS database or directly from participants.

In this revised version, we added all the above-mentioned details raised by the reviewer in the methods section related to the EEG datasets description. In addition, there is now a preprint available which describes the data collection and preprocessing for the present study (Gilbert et al., 2025), which is now cited and provides further detail.

7. Ethics and Participant Information

There is no mention of ethical approval, informed consent, or participant demographics (age range, gender distribution, inclusion/exclusion criteria). Such information is essential for studies involving human EEG data and should be explicitly reported.

Both studies were approved by the University of Florida's institutional review board and conducted in compliance with the Declaration of Helsinki. Participants gave written consent to participate in the studies and course credits were given as compensation. Now, we added a mention to these points in the methodology section.

8. Model Transparency and Ablation Analyses

The model description, while conceptually clear, lacks sufficient technical specificity. The number of filters, layer sizes, stride parameters, and output dimensions are missing or, in some cases, only briefly mentioned, which limits reproducibility. Moreover, it is unclear which architectural components are critical to performance. An ablation analysis (e.g., removing the conditioning layer or varying latent dimensionality) would help demonstrate which aspects of the architecture meaningfully contribute to model accuracy and generalization.

We agree with the reviewer that the model description was insufficient detailed in terms of parameters. More technical details have been added to the manuscript.

Regarding the criticality of the architectural components to the performance, we have expanded their overall description and illustration. Although all major components are needed for performance, their settings and dimensionality greatly affect performance and we have added additional information regarding these settings and how they were chosen. For example, we decided to use 3 convolutional layers for both the encoder and decoder

subcomponents and a latent space or bottleneck of 256 neurons. We provided some exploration of this space using the simulation/manipulation approach outlined above in comment 1. Systematic ablation analyses for these components are possible and could be included in the manuscript, but at this time appeared to lengthen the manuscript and supplementary materials beyond what may be desirable. If the editor and reviewer find these analyses helpful however, we are happy to included them.

9. Semantic and Emotional Labeling

The paper should clarify how "semantic" and "emotional" content were quantified. Were labels derived automatically from CLIP embeddings, or based on pre-existing IAPS ratings? If the latter, emotional valence and arousal can differ substantially from participant-reported experience. Explicitly defining these constructs and their sources is necessary for scientific transparency.

This is an important point. We have attempted to expand and clarify our description in the manuscript. The semantic content was quantified using CLIP embeddings into a 512 multidimensional vector. Thus, the Concept2Brain model works with CLIP embeddings and emotional ratings are not used for training. The pleasant, neutral and unpleasant categories were derived from standardized/normative ratings available for the pictures and validated against the ratings given for each picture by the 88 participants in the training data set. For the validation studies, we examined the extent to which the synthetic data generated by Concept2Brain show strong and well-established modulations by emotional content, including the late positive potential (LPP) component (Pastor et al., 2008), or the N170 (Schindler et al., 2023). The revised version of the manuscript describes the distinction between semantic processing and emotional identification more systematically, and uses a different organization to distinguish training input from labels used for validation and generalizability studies.

10. EEG Preprocessing and Referencing

Key preprocessing details are missing or ambiguous. There is no information about referencing schemes after interpolation (e.g., average reference or Cz reference), baseline correction procedures, or criteria for trial exclusion. These choices have a significant impact on the resulting ERPs and on how well synthetic data can be compared with real EEG.

A detailed description of pre-processing steps is given now under “Training the Concept2Brain model”, subsection “EEG data sets”.

11. Cross-Validation and Generalization

There is no clear cross-validation or hold-out testing strategy. A clear validation scheme (e.g., leave-one-participant-out or leave-one-stimulus-out) would provide stronger evidence of the model’s robustness.

We agree. The original approach relied on using all data to train the Concept2Brain model and offer validation and generalization demonstrations by using out-of-sample analyses. However, after considering all reviewers’ comments, we implemented cross-validation analyses as recommended.

Given the nature of the cVAE, we cannot conduct a leave-one-participant-out validation. In this scenario, the information of one subject would be held out and never shown to the model; as a consequence, we could not predict this participant’s EEG signal. Therefore, we decided to employ a hold-out picture strategy, using EEG trials from all participants from 324 pictures for training and tuning. Specifically, 288 pictures (80%) were used to train the cVAE and then used to train the cross-domain model. To prevent overfitting of the cross-domain network, 36 pictures (10%) served to monitor the training process and to prompt early-stopping if the loss function began to rise. Afterwards, the trained architecture was frozen and data from 36 pictures (10%), unseen during training and monitoring, were used to measure out-of-sample validity. Thus, validation was based on EEG signals of corresponding empirical and synthetic participants in response to the same pictures. Pearson’s correlation coefficient was computed across pictures, time, and topography. This procedure was repeated 10 times (10 folds) rotating through the 10% of the held-out pictures, and ensuring that all of the 360 pictures eventually served as part of the validation set.

Results showed a general high similarity between real and synthetic data, demonstrating the capability of the Concept2Brain model to generalize to new stimuli. A new section describing this cross-validation analysis has been added in the Results section of the manuscript. In addition, more out-of-sample validations using datasets like THINGS-data or ERP CORE have been added to the manuscript, complementing the above cross-validation results.

12. Model Complexity and Risk of Overfitting

Given the relatively small dataset (~13,600 trials) and the apparent size of the neural architecture, there is a significant risk of overfitting. The paper does not mention the use of regularization methods such as dropout, early stopping, or weight decay. Including these details—and ideally reporting training and validation loss curves—would help assess whether the model truly generalizes beyond the training data.

These are very pertinent concerns. We now describe in greater detail the architecture's properties as well as the intensive training (1,300 epochs) in which the VAE learns to represent the electrophysiological activity in its latent space (see Figure 2B). We followed the recommendation of showing validation and training curve data for both the cVAE training and the cross-domain network. To avoid overfitting of the latter component, we designed it to be a small network (3 layers) translating the 600 vector (512 CLIP + 88 participants) into a 256 vector (the electrophysiological latent space). To prevent the network from overfitting, we used a subset of the data (the evaluation dataset) unseen in the training phase. In each epoch, a loss function was calculated for this set, early stopping the training process if the evaluation loss was increasing (patience = 30) and adopting the best model (see Figure 2C, orange line). During the creation of the architecture, we did not see significant enhancement of the generalization due to dropout or weight decay strategies, so they were discarded for the final model.
In summary, the new version of the manuscript provides the requested details. Figure 2 now illustrates the loss functions during the training phases of both the VAE and cross-domain networks.

Reviewer #1 (Remarks on code availability):

The code is available on github and, when downloaded, appears to function well.

We appreciate reviewer's time to evaluate the manuscript and the insights and thoughtful feedback. Clearly, it has deeply contributed to enhance the work and provide more evidence and robustness to the model.

Reviewer #2 (Remarks to the Author):

This paper purports to provide "a computational architecture for converting information contained in a text or image into synthetic ERP waveforms." The model and its evaluation do not, however, live up to these claims.

The model is trained on a total of 360 images. This is extremely insufficient to claim that the model is suitable as a general EEG encoder. The amount of data also appears insufficient to learn to approximate individual subjects given that an individual actually only has data from 120 or 240 images.

We share the reviewer's concern and have expanded on our analyses and provide information regarding the technical steps we took to demonstrate the validity of the architecture. In the revised text, we also discuss its limitations and emphasize its potential for expansion and growth after adding additional training data.

First, one major objective of the Concept2Brain model was to generate synthetic datasets that contain virtual participants. Specifically, we aimed to create a tool for simulating brain activity in a substantial human sample, large enough to capture typical inter-individual variability (in our case 88 participants). The use of a larger sample allows users to estimate and quantify variability, e.g. in in waveform shape and topography, helpful for planning empirical studies and for understanding the simulated signal, and for quantifying robust tendencies in the data, present across different observers. Currently, there are rich datasets like THINGS-data (Hebart et al., 2023) including a large amount of trials and pictures up to 1,854 concepts (Hebart et al., 2019). However, these datasets (both MEG and fMRI) only contain few human participants (4 and 3, respectively). Thus, their capability for simulating the outcome of empirical studies with typical participants is reduced. Alternatively, datasets like Grootswager et al. (2022) or Gifford et al. (2022) balance the sample size (50 and 10, respectively) with the number of exemplars (1,854 and 1,654, respectively). They do so however by minimizing presentation duration and trial length, reducing the ability to examine the unfolding of neural activity over time, and thus attenuating sensitivity to late, higher-order cognitive, motivational, or emotional processes.

A second point that is now discussed in the manuscript is that the well-curated complex scenes used in this study provide rich information about multiple scene elements instead of representing a narrowly defined object. Therefore, each one of the 360 pictures used to train the model contains information about more than one concept, stimulus dimension, or feature. Together with the participant variability in the training data and the properties of the

cVAE (below), this enriches the training set and contributes to the valid reproduction of ERP components seen in out-of-sample analyses.

We also expand on how the variational autoencoder facilitates generalizability. It generates, through Bayesian updating and regularization across participants and exemplars, distributional representations of the data that most closely match the empirical distributions. These properties are now detailed in the revised manuscript.

As recommended, we also conducted two new validation analyses. (1) A 10-fold cross-validation with 36 held-out pictures (see next comment below). (2) Out-of-sample analysis were conducted using the above-mentioned THINGS and ERP CORE data sets. In these analyses, the synthetic EEG responses to hundreds of inputs showed similar behavior as the empirical signals.

The authors do not show training curves for the model or report the train or test MSE, making it hard to evaluate the model's performance directly. Furthermore, it is not clear how the train/test split was done. It seems that it is *not* the case that certain images were held out entirely, but rather the data was arranged as subject-image combinations and these were randomly split. This represents only the most minimal test of generalization, and even the results on this test set were not shown. Validation data is not discussed at all. The ability of the model to replicate specific individuals is also not analyzed.

As far as I can tell, the results in Fig2A-D are evaluated primarily on training data (it seems that all images were used for these analyses, meaning that 80% of the data used is training data). The performance of the model on training data is not relevant or appropriate to report; it is not indicative of the model's general abilities (and even when evaluating on training data the model still doesn't seem to generally correlate well with the real EEG, though this is not fully reported). Fig 2E appears to actually use images not trained on, but the actual fit of the model to EEG data is not reported and the correlation to the arousal metric is quite weak. Fig 4F-G appears to use non-training images, but again does not report fit to EEG data, but rather demonstrates a simple qualitative finding from the literature. Therefore these results do not help support the idea that this model has learned a general ability to predict EEG data in response to any image.

We agree with this comment. The original approach relied on using all data to train the Concept2Brain model and offer validation and generalization demonstrations by using

out-of-sample analyses. However, after considering all reviewers' comments, we implemented cross-validation analyses as recommended.

Given the nature of the cVAE, we cannot conduct a leave-one-participant-out validation. In this scenario, the information of one subject would be held out and never shown to the model; as a consequence, we could not predict this participant's EEG signal. Therefore, we decided to employ a hold-out picture strategy, using EEG trials from all participants from 324 pictures for training and tuning. Specifically, 288 pictures (80%) were used to train the cVAE and then used to train the cross-domain model. To prevent overfitting of the cross-domain network, 36 pictures (10%) served to monitor the training process and to prompt early-stopping if the loss function began to rise. Afterwards, the trained architecture was frozen and data from 36 pictures (10%), unseen during training and monitoring, were used to measure out-of-sample validity. Thus, validation was based on EEG signals of corresponding empirical and synthetic participants in response to the same pictures. Pearson's correlation coefficient was computed across pictures, time, and topography. This procedure was repeated 10 times (10 folds) rotating through the 10% of the held-out pictures and ensuring that all of the 360 pictures eventually served as part of the validation set.

Results showed a general high similarity between real and synthetic data, demonstrating the capability of the Concept2Brain model to generalize to new stimuli. A new section describing this cross-validation analysis has been added in the Results section of the manuscript. In addition, additional out-of-sample validations using datasets like THINGS-data or ERP CORE have been added to the manuscript, complementing the above cross-validation results.

Regarding the reviewer's comment about model replicability for specific individuals, we agree that it was merely shown but not deeply analyzed in the initial version. Now, differences between real and synthetic evoked responses are shown for the very first 3 participants (see Figure 3 E). They illustrate how well the synthetic time series closely fit the empirical data. In addition, we estimated an correspondence matrix (Figure 3 D). This matrix contains Pearson correlations between the empirical and synthetic EEG signal for each empirical participant. Consequently, it informs on how much data from pairs of participants (empirical and synthetic) correspond. Here, our correspondence matrix shows the highest correlation at the diagonal for all the participants, thus indicating high correspondence between empirical and synthetic participants.

The authors refer to the text-based simulation as "replicating an entire experiment", but it is not a replication if the experiment was never done, and indeed none of the results are compared to any text-based experimental results. Rather, the model outputs are qualitatively compared to image-based experiments. Given this and the nature of CLIP, it seems that this model would not do well in predicting how EEG signals differ when text vs images are used, and therefore it is not a good encoder model for EEG.

This is another well-taken comment. We indeed never “replicated” the experiment mentioned, and the language used in the prior version was confusing. In the present version, we instead simulated replications of two public data sets by passing these study’s stimuli into the Concept2Brain model: First, we used the THINGS-data (Hebart et al., 2023), feeding the Concept2Brain model with the pictures from the THINGS dataset (Hebart et al., 2019). Results (new Figure 4 of the manuscript) exhibited strong correlations between the published data and the Concept2Brain output. In the case of MEG activity, key electrophysiological components were replicated in the simulated data, and the BOLD similarity matched latencies and topographies.

Next, we reproduced the N170 subset of ERP CORE data set (Kappenman et al., 2021). Stimuli were pictures of 40 cars and 40 faces. Again, the original stimuli were fed into the Concept2Brain model and the empirical (ERP CORE) and synthetic (Concept2Brain) data compared. The typical electrophysiological differences between face and car stimuli were present in both datasets.

These two out-of-sample validations were included in a new section in the Results. The example with written text as input is used as an example of potential usage instead.

Fig 5 shows that affect can be decoded somewhat from the simulated EEG signals. It is unclear what this indicates about the model as these results are not directly compared to real data. The absolute decoding performance is not indicative of the quality of the EEG model specifically because it is probably the case that affect could be just as well if not better decoded from CLIP output alone.

We understand reviewer's concern and agree that affect is likely to be decodable from the CLIP output alone. However, the intention of the decoding analysis presented in the Discussion section is not to demonstrate the capacity of CLIP's output, but to demonstrate that the electrophysiological latent space is better decodable than the EEG signal. Far from being a mere decoding benchmark, it is intended to show the strength of the VAE to capture and refine particular and relevant features such as affective valence from

electrophysiological signal, being the central core of the Concept2Brain architecture. As suggested, the text has been shortened to clarify the intention of the figure (now Figure 7).

In total, the model, when not evaluated on training data, shows some qualitative similarity to EEG signals specifically around affect. This does not constitute a "an AI model for predicting neurophysiological responses to text and pictures" in any general sense. The authors claim that through the model "differences can be examined at the level of individual prompts and individual participants, without the need of large datasets" and that "in clinical studies, this model may be used to generate typical brain responses for initial comparisons with a patient group". There is no evidence provided to support these claims and in fact the latter would be incredibly inappropriate and potentially damaging.

We have removed language suggesting usage in clinical studies. We believe that the additional analyses with various out-of-sample data support our suggestion that the model is useful for simulating electrophysiological response to a wide range of stimuli, including objects, faces, and naturalistic scenes. We have however discussed limitations and the need for future expansion, in the discussion.

Authors would like to thank the reviewer for its time employed in this review and the stimulating comments. We hope that this new version of the manuscript answers its concerns.

Reviewer #3 (Remarks to the Author):

This paper presents a methods to generate visual ERPs from images (and other content) using a variational autoencoder and a connection to OpenAI CLIP platform. Overall I think it's a useful contribution, but I have some comments and suggestions.

1. Presentation: My main critique of this paper is that the presentation of the text is quite suboptimal. At the moment, if feels the flow is rather disorganised, forcing the reader to go back and forth to understand things:

-> The Results assume the reader knows things that are only explained later. As an example, please provide *conceptual* details on the conditioned Variational Autoencoder, the electrophysiological latent space, and the cross-domain neural network, as well as anything that is needed to get a basic understanding of the Results. The purpose and meaning of these have to be clear to a non-technical reader.

Thank you for this helpful comment. We have rewritten the manuscript to follow these recommendations. Adding several cross-validation procedures and examples also facilitated descriptions of the underlying concepts and methods. We hope that this will facilitate reading.

-> Datasets 1 and 2 are not explained until much later on, which doesn't help.

The new version briefly describes the datasets before detailing results. An in-depth description is still provided in the methods.

-> In Fig2, the authors first talk about panel A, then E, then BCD. And from then they jump to Fig6. It would be a good exercise to try to describe the figures sequentially, I think this would improve the flow substantially.

The panels are now provided in appropriate order.

-> Some results (fig5) are presented in Discussion instead of Results which it's quite unorthodox and also a bit confusing.

We re-organized the results. Merely the latent space analysis is still provided as part of the discussion because it is concerned with interpretation and future directions.

These are just examples: in general please do a careful read of the whole manuscript.

We completed several passes with the helpful suggestions regarding readability in mind. We had several readers and collaborators provide edits. We hope we have succeeded in making organization and content more accessible.

2. Subject- vs. group-level results: I think a very important panel for the validation of this paper is 2D because it's most quantitative result showing that this works. If every dot is a time point / subject, it would be very useful to use colours to indicate time, and indicate the correlation in the Figure. Also, this aggregates all subjects in a single measurement. What about correlations for each subject? It would be useful to show a distribution of those, perhaps across all time points. I understnd the point of this is group-level results, but in general, I'm missing subject-specific results, which are also important scientifically.

The revision now provides a complete participant-level analysis and illustrations of individual participant-level predictions. We added an correspondence matrix based on Pearson correlations (see Figure 3 E). It quantifies the similarity between the EEG activity of each empirical participant and the corresponding synthetic participant. The diagonal elements of this matrix contained the highest correlation indices for each row, indicating that the data from the empirical participants matched their synthetic counterparts.

We also now show averaged EEG activity from electrode Oz for the first three participants in Figure 3 D. This figure also illustrates the pronounced correspondence between data from matching empirical and synthetic participants.

3. Validation: Overall, the authors showed a set of cases where the model produces responses that make sense in one way or another. I would guess these were not the first and only tests that the authors have tried, and that other things didn't work - which is absolutely normal and to be expected. Now my question is: how often does it work? When it didn't work? It would be very useful to know the limits of the model as well. In this line, for example, what would it happen in the result shown in Fig2D if the model is trained on a subset of the subjects and tested on unseen subjects? (I understand the scatterplot is from subjects that were used in both train and test). Of course the latter results are shown for data that was not used in training, but they are rather qualitive and cherry-picking on very specific characteristics of the ERP. Would an equivalent of Fig 2D work for unseen data?

This is a great question. The revised version of the manuscript includes additional validation analyses. First, the 10-fold cross-validation within the original EEG dataset indicates the capability of the model to predict individual pictures responses. Interestingly, one of the pictures showed a significantly lack of correlation between synthetic and empirical EEG signal (Pearson's correlation of -0.1, Figure 2D). This picture from the International Affective Picture System shows a traditionally attractive person with long hair and a wet t-shirt on a beach, conveying erotic innuendo, and rated as pleasant by the participants in the normative IAPS sample. The CLIP encoder is known to not reproduce complex erotic content. This illustrates a shortcoming of the current model configuration, which is now reported in the manuscript (Figure 2D and text below).

Second, the Concept2Brain model's output to hundreds of concepts were compared with public MEG and fMRI data sets. This out-of-sample validation, replicating empirical data demonstrates the model's validity. Overall, it showed strong correlations between the predicted EEG data and electromagnetic and hemodynamic recordings. In both cases, all

stimuli available in the empirical datasets (1,854 for MEG and 720 for fMRI) were used, thus preventing effects of picture selection.

Although the participant-level conditional autoencoder architecture prevented us from conducting out-of-sample analyses with held-out participants, the revised version contains a more balanced and more quantitative description of the model's strengths and weaknesses, hopefully addressing the spirit of these well taken comments.

4. Beyond the averages: Do the authors have any comment about aspects that go above and beyond the ERP average? There is a lot of information at the trial basis, and other simulation methods, like Genephys (Vidaurre, 2024 *) work at the level of trials instead of averages. What would be the pros and cons of each approach with respect to each other? In general, disentangling the different contributions to the effect requires having trial-basis signals (Vidaurre, Cichy, Woolrich; 2021 **), which I would understand as a limitation of Concept2Brain. Of course, I understand capturing between-trial variability is beyond scope, so I'm only asking to discuss this.

* https://elifesciences.org/articles/87729

** https://academic.oup.com/cercor/article-abstract/31/12/5664/6324865

(Apologies here for citing my own work, it's just the things that come to mind)

We agree that this is a very relevant topic. The newly added analysis of oscillatory content (Figure 3D) demonstrates that some information from the individual trials, such as aspects of induced oscillations, is contained in the synthetic data. However, because of the properties of the variational autoencoder (VAE), the individual synthetic data segments possess higher signal-to-noise than empirical single trials and thus cannot replicate all aspects of empirical single trials, as can other platforms such as Genephys (Vidaurre, 2024).

In the revised manuscript, we acknowledge this fact and emphasize that the lack of single-trial variability in response to the same stimulus represents a problem for many advanced single-trial analyses.

5. Section "Electrophysiological response based on emotional text": I don't quite get the scientific use of this. The proposed model is about generating responses to visual stimulation. The fact that CLIP can draw links between text and images seems beyond the merits and capacities of the proposed model, other than a practical convenience if the user do not have images at hand; but, again, other ready-to-use tools exist for that. Please clarify.

The new version of the manuscript now emphasizes the validation of the model and its use with picture input. As the reviewer noted, there may be some situations where no pictures are available, or where the generation of pictures is not feasible. In our own experience, there is also a loss of information when translating complex semantic content into one picture using current encoding models. We thus leave this as an option, as it offers convenience and accessibility for some users. As recommended, the revised version of the manuscript however de-emphasizes the importance of the text input.

6. Section "ERP generation: Web implementation": I find the section slightly confusing. As I understand, the web implementation differs in the interface (web vs non-web), but the engine is essentially the same. Why does it then need a separate demonstration with another set of stimuli, resulting ERPs, etc? Overall, Fig4 mixes another "scientific demo" (alligator, desk and beach) with showing that it also runs on web - but you don't need another set of stimuli for that. If this new set of stimuli does bring something new to the story, or even if it just offers further empirical validation, but there's nothing specific about being run through the web, I would put it with the other demonstration and just say that Concept2Brain can run on the web.

We agree that this section was confusing and used unnecessary space of the manuscript. One of the useful features of the Concept2Brain model lies in its accessibility and ease of use. The revised version attempts to describe the use of the web resources without taking up too much space and without confusing readers with conflicting information.

7. Example on conceptual content in Fig 4D. Following on the comment above, the fact that the pipeline can generate ERPs from complex sentences isn't surprising given that CLIP can basically link anything to anything. So the authors' approach will always generate something anyway. The more interesting question is why it can generate an effect between this negatively arousal sentence and another that is more neutral or even positive: To which

images in the training data set on which Concept2Brain was trained this is being linked to? (This is also confusing to be mixed with the web feature by the way.)

We agree that this portion may be confusing, as it seemed to be unique to the web version. We have retained the example as an application that demonstrates that the model generates expected findings with stimulus material that does not correspond directly to any individual picture in the training set, but represents a mixture of contents represented across a variety of complex scenes used in training. It also suggests that the motivational/emotional connotation was also implicitly learned by the architecture, without being given emotion labels or semantic descriptions. To address this valid concern however, we de-emphasized this example and its implications in the revised text.

Minor / detail comments:

- "Instead of decoding brain activity to classify or reconstruct a given stimulus, event, or condition, this approach intends to encode the stimulus to generate bio-realistic brain activity."

It would be useful to make the distinction of models to predict brain activity using features from the stimuli from actual simulation methods to actual simulate brain data given a set of hyperparameters.

- Gifford AT, Bersch D, Roig G, Cichy RM. The Neural Encoding Simulation Toolkit.

We thank the reviewer for highlighting this important distinction.

We concur that encoding models predict neural activity from stimulus features, whereas simulation methods generate activity from internal biological mechanisms. In our work, images are the input: a CLIP model (or any suitable alternative) extracts a conceptual representation, which a VAE then uses to infer EEG activity. Concept2Brain is a generative neural-encoding model that predicts electrophysiological responses from high-level semantic representations of stimuli. Although it produces synthetic EEG, it is not a simulator based on internal hyperparameters or bio-plausible mechanisms such as HNN (Neymotin et al., 2020), spiking neural networks (Yamazaki et al., 2022), or models like Jansen-Ritter.

Regarding the Neural Encoding Simulation Toolkit (NEST), we have not found a peer-reviewed reference; the only source is its creator's website: https://www.alegifford.com/projects/nest/. NEST serves a similar purpose to Concept2Brain

and, based on its repository, dataset, and AI model, appears promising and likely outperforms Concept2Brain in reconstructing early ERP components, particularly given NEST's use of a large dataset of short (100 ms) stimuli. However, our focus is on contextualized images and late-component responses. Here, CLIP embeddings provide a richer abstraction of not only object features but entire scene representations. Concept2Brain therefore offers a complementary approach for studying later components with contextual input. While NEST predicts activity directly, we employ a variational autoencoder that captures latent electrophysiological information, enabling the generation of new tracks with improved generalization (Abdi-Sargezeh et al., 2025; Mendonça et al., 2023). It successfully reproduces data for all 88 participants. Additionally, we provide a cloud-computing platform that allows users to run the tool without installation or hardware requirements.

The text highlighted by the reviewer has been updated accordingly, and the comparison with other simulation models is now included in the Discussion section.

- "If information is systematically shared between real world concepts expressed in media and text (conceptual latent space in Figure 1.2) and their representation in the human brain"

There's quite a leap from the previous text in the paragraph to this statement. This is the first occurrence of "media and text". At this stage it is unclear what media and text -and in general, language- have to do with ERPS/encoding/decoding methods in Neuroscience. Please clarify: what is the rationale, assumptions, motivation, etc of such link?

This paragraph has been rewritten and restructured to provide a more seamless description of the argument.

- LPP: define in the first use (it says Late Positive Potential component a few lines below it's used for the first time). Both in caption and main text

This has been corrected.

- Fig 2BC, add legend for colours

Thank you. New Figure 5 has a legend.

- Caption 2. "normative arousal ratings collected in a standardization sample". Some more explanation is needed to understand what this refers to.

The revised version gives a brief explanation of where normative arousal ratings come from.

- Fig 2E: The explanation seems insufficient, both in main text and caption

The explanation has been expanded.

- "showed a statistically significant correlation of r = 0.23". Be clear between what and what.

We have specified this and other similar analyses.

Reviewer #4 (Remarks to the Author):

This study is evaluated on a modest image dataset:

- 45 subjects watching 120 pictures, and

- 43 subjects watching 240 images:

This is substantially lower than previous studies:

- Teichmann et al and Hebart (MEG): 10 subjects 22k images

- Grootswagers et al (EEG 2022): 50 subjects watching 22k images

- Gifford et al (EEG 2022): 10 subjects watching 16k images.

This small dataset means that the model can only learn the brain responses to a small diversity of image. Consequently, it would, even in principle, be difficult for the model to generalize to a large set of naturalistic images / prompts.

We thank the reviewer for raising this important point. In the revised version of the manuscript, we have addressed some of these concerns and have explained the limitations but also the unique contributions of Concept2Brain with respect to the issues raised in this reviewer comment. For ease of reading, these points are summarized in the following paragraphs.

First, the main objective of the Concept2Brain model is to generate new synthetic datasets high in realism and capable of reproducing aspects of inter-individual variability in waveform and topography. We also aimed at synthesizing realistic waveforms, representing the full temporal unfolding of electrophysiological responses to input. Existing approaches have used few observers and/or very short rhythmic presentations of many stimuli, to increase the size of the training set. This gain is however at the cost of temporal fidelity: Although deconvolution techniques can be used to estimate individual visuocortical responses to pictures presented during rapid serial presentation (RSVP; see e.g., Grootswager et al. 2022 or Gifford et al. 2022), the RSVP approach attenuates and obscures late event-related

responses—shown extensively in careful, decade-long work in the ERP/RSVP/SSVEP literature. Thus, the present approach contributes inter-individual variability and thus heightened bio-realism, combined with the unique ability to generate unadulterated waveforms spanning hundreds of miliseconds, representing early and late responses, as studied in ERP research of the P300 and Late Positive Potential and critical for higher order cognition.

Second, the present approach involved complex naturalistic scenes instead of isolated objects. Naturalistic scene pictures result in a more variable sensory encoding, while also conferring information needed for contextual, higher-order semantic and affective categorization (Bo et al., 2022; Farkas et al., 2025; Frank & Sabatinelli, 2017; Seijdel et al., 2021). Additionally, the complex scenes tend to contain multiple distinct elements one single image. Therefore, each one of the 360 pictures used to train the Concept2Brain architecture using the CLIP model may represent more than one concept. As a result, the naturalistic scene approach enriches the training set and provides more than 360 concepts.

The Concept2Brain model utilizes a conditioned Variational Autoencoder (cVAE) architecture trained using Kullback-Leibler loss as a regularization method to force the latent dimensions into a normal distribution. In this architecture, electrophysiological information is stored in a sparse fashion across the 256 bottleneck neurons. This sparsity makes the model more robust by forcing the decoder to integrate global activation patterns of the bottleneck to translate the latent representations into bio-realistic EEG signals. The VAE facilitates generalizability by generating, through Bayesian updating and regularization across participants and exemplars, distributional representations of the data that most closely match empirical distributions. By capturing the underlying semantic and emotional structure of the input, the architecture can successfully map novel CLIP embeddings into the latent electrophysiological space, enabling the generation of realistic waveforms for unseen images or prompts.

To illustrate this capability, we have added two new main analysis in the present version of the manuscript: (1) A 10-fold cross-validation with 36 held-out pictures indicates the resemblance between real participant's EEG signal and its synthetic counterpart for these images (see next coment below). (2) Out-of-sample analysis were conducted using the above-mentioned THINGS-data with MEG and fMRI data. Crucially, the brain's response to hundreds of concepts showed similar behavior than the synthetic EEG signal generated by the model, both at the magnetic field and BOLD activity. Two new Results' sections has been added to apport evidence on model's robustness and generalization capability. Taken together, results supports evidence on generalization capabilities of the Concept2Brain model to generate brain's response to new stimuli based on the 360 pictures.

Method:

The train/test method may be partly invalid: The authors indicate that the training set is 80% of the EEG data (10K trials).

However, the (120 or 240) images are repeated across participants.

To ensure that the model does not learn the association between EEG and images "by heart", it is necessary to perform these experiments with out-of-image predictions (as in Allen et al 2022).

For example, if we assume that there is an EEG component shared across participants (e.g. the famous N170 specific to faces), then the model could learn that for images A, B, C, it should predict a N170. When evaluated on (held out) EEG responses to the same A, B and C images, it would then be above chance.

Specifically, the authors need to do a group splitting based on the image at line:

https://github.com/ASantosMayo/Concept2Brain/blob/main/Concept2Brain_training_script.py#L178

This is enormously helpful, thank you for reviewing the code and for this suggestion. The original approach relied on using all data to train the Concept2Brain model and offer validation and generalization demonstrations by using out-of-sample analyses. However, after considering all reviewers' comments, we implemented cross-validation analyses as recommended.

Given the nature of the cVAE, we cannot conduct a leave-one-participant-out validation. In this scenario, the information of one subject would be held out and never shown to the model; as a consequence, we could not predict this participant's EEG signal. Therefore, we decided to employ a hold-out picture strategy, using EEG trials from all participants from 324 pictures for training and tuning. Specifically, 288 pictures (80%) were used to train the cVAE and then used to train the cross-domain model. To prevent overfitting of the cross-domain network, 36 pictures (10%) served to monitor the training process and to prompt early-stopping if the loss function began to rise. Afterwards, the trained architecture was frozen and data from 36 pictures (10%), unseen during training and monitoring, were used to measure out-of-sample validity. Thus, validation was based on EEG signals of corresponding empirical and synthetic participants in response to the same pictures. Pearson's correlation coefficient was computed across pictures, time, and topography. This

procedure was repeated 10 times (10 folds) rotating through the 10% of the held-out pictures and ensuring that all of the 360 pictures eventually served as part of the validation set.

Results showed a general high similarity between real and synthetic data, demonstrating the capability of the Concept2Brain model to generalize to new stimuli. A new section describing this cross-validation analysis has been added in the Results section of the manuscript. In addition, additional out-of-sample validations using datasets like THINGS-data or ERP CORE have been added to the manuscript, complementing the above cross-validation results.

Results

First, there are several perfectible statements : “For example, the simulated ERP at the mid-occipital Oz electrode shows deflections consistent with early visuo-cortical responses to stimulus onset, whereas the simulated data at the mid-parietal Pz electrode contains the late positive potential component, modulated by emotional content. Also consistent with empirical data, the ERP waveforms show pronounced differences across participants.”

-> These elements are not thoroughly evaluated with statistical methods.

We thank the reviewer for his comment. The new version of the manuscript has been updated throughout to include quantitative indices such as Pearson correlations when evaluating similarity and dissimilarity.

Regarding the differences between participants, we have conducted a participant-level analysis (see Figure 3 E). It quantifies the similarity between all pairs of empirical and synthetic participants using Pearson’s correlation. Maximal correlations for matching empirical-synthetic participants were found along the diagonal of this matrix, indicating that the architecture reproduced distinctive participant-level features inherent in the waveform and topography of the electrophysiological responses.

Second, the main validations of the models stand on two main results:

1. LPP components correlate with the normative arousal ratings for each picture.

2. A modulation of the prediction depending on the presence of a face.

While interesting, these two elements are quite limited compared to the scope of building a predictive model of EEG responses to natural images.

In response to this well-taken comment, we have added extensive cross-validation and out-of- sample analyses with a variety of examples, including reproducing effects of faceness versus objectness, facial expression, emotional content, from the present and other publicly available data sets.

First, the 10-fold cross-validation within the original EEG dataset indicates the capability of the model to predict individual pictures responses. Interestingly, one of the pictures showed a significantly lack of correlation between synthetic and empirical EEG signal (Pearson's correlation of -0.1, Figure 2D). This picture from the International Affective Picture System shows a traditionally attractive person with long hair and a wet t-shirt on a beach, conveying erotic innuendo, and rated as pleasant by the participants in the normative IAPS sample. The CLIP encoder is known to not reproduce complex erotic content. This illustrates a shortcoming of the current model configuration, which is now reported in the manuscript (Figure 2D and text below).

Second, the Concept2Brain model's output to hundreds of concepts were compared with public MEG and fMRI data sets (THINGS-data, Hebart et al.,2023). This out-of-sample validation, replicating empirical data demonstrates the model's validity. Overall, it showed strong correlations between the predicted EEG data and electromagnetic and hemodynamic recordings. In both cases, all stimuli available in the empirical datasets (1,854 for MEG and 720 for fMRI) were used, thus preventing effects of picture selection.

Next, we reproduced the N170 subset of ERP CORE data set (Kappenman et al., 2021). Stimuli were pictures of 40 cars and 40 faces. Again, the original stimuli were fed into the Concept2Brain model and the empirical (ERP CORE) and synthetic (Concept2Brain) data compared. The typical electrophysiological differences between face and car stimuli were present in both datasets.

These novel cross-validation and out-of-sample validations were included in a the Results section of the new version.

Contributions:

The authors introduce a new encoding model, but do not compare it to baseline models e.g. linear TRF, or deep encoder etc (e.g. Gifford et al 2022, Benchetrit et al arxiv 2025, O Chehab, Defossez et al 2022).

We now discuss these models in greater detail, some of which we were unaware of--thank you again for this helpful comment. As stated above, we now more explicitly state that the Aim of Concept2Brain is to generate new EEG datasets of 88 participants, providing individual waveforms with high fidelity, including in late temporal regions. We have added two paragraphs that compares the resulting key differences between the current and prior models.

Minor:

- The introduction could be substantially shortened: e.g. by perhaps limiting the historical perspective of the invention of EEG and its various use cases, and the explanation of what an evoked response is.

In the new version, the introduction has been modified and streamlined by rewriting and by removing several parts of the text, while preserving the necessary background.

- The architecture design are not motivated: the authors should provide ablation experiments to evaluate the utility of their choices (number of layers, multi stage fitting etc)

The revised version contains additional details and rationale for the architecture design. For example, in the case of the autoencoder bottleneck, we decided the number of neurons (latent dimensions) based on initial tests exploring other configurations, which are now described, accompanied with additional descriptions throughout. Furthermore, information and graphical representations of the application of loss functions during the training phase (see Figure 2 B-C) has been included.

Additionally, the revised manuscript includes Supplementary Information 1, describing interpretability analyses conducted on the VAE architecture to disentangle its “black box” nature, resulting in a better understanding of the architecture and its design.

- “However, despite their potential, encoding models for EEG remain relatively under-explored, with many questions regarding their ability to accurately replicate the complexity of real brain activity.”

-> It would be great to detail further these remaining questions to clarify the contributions.

We provide a more detailed description of the remaining questions and how Concept2Brain addresses them. As stated above, these include participant variability and temporal unfolding of the event-related waveform, but also oscillatory content.

- Pearson's correlation r = 0.640, p-value < 0.01

-> Given the strength of this correlation, and following Fig2E caption, I understand that this statistics is performed on the grand ERP average across subjects. However, a valid statistics would require being tested across the 88 subjects, since the other dimensions (channels, times, etc) are not independent from one another

We understand the concern pointed out by the reviewer and apologize for the lack of clarification in the previous version of the manuscript. The strong correlation remarked by the reviewer (Pearson's correlation r = 0.640, p-value < 0.01) corresponds to a data recovery analysis carried out with all the data. This means that each of the black points shown in the figure (now Figure 3 C) corresponds to one EEG value from any one of the 129 channels, one of the 750 time points and one of the 88 subjects. Therefore, the scatter plot shows 8,514,000 black points (129 * 750 * 750). Here, our goal is to show a global data recovery analysis whereby all the predicted and real EEG values can be shown in one plot, and Pearson's correlation index calculated.

In addition, we agree with the reviewer's suggestion to carry out the analysis across the 88 subjects. This analysis is shown in Figure 3 B. Here, a Pearson's correlation index was independently estimated for every channel and time point, using the 88 subjects as distribution for the calculation. This analysis highlights which areas and time latencies showed the highest similarities between the synthetic data and the empirical EEG recordings. Supporting the sensitivity to semantic content, the topography and latencies of the Pearson correlations revealed larger similarities between synthetic and empirical brain activity during the late positive potential across the parietal areas, where these late potentials are typically maximal.

I conclusion, we would like to thank the reviewers for their careful reading and helpful comments. We hope that the revisions and new analyses provided help to clarify the points raised and support the usefulness of Concept2Brain for the community.

REFERENCES

Bo, K., Cui, L., Yin, S., Hu, Z., Hong, X., Kim, S., Keil, A., & Ding, M. (2022). Decoding the temporal dynamics of affective scene processing. *NeuroImage*, *261*, 119532. https://doi.org/10.1016/j.neuroimage.2022.119532

Farkas, A. H., Gehr, M. C., Jia, H., & Sabatinelli, D. (2025). Measuring Realistic Emotional Perception With EEG: A Comparison of Multimodal Videos and Naturalistic Scenes. *Psychophysiology*, *62*(1), e14765. https://doi.org/10.1111/psyp.14765

Frank, D. W., & Sabatinelli, D. (2017). Primate Visual Perception: Motivated Attention in Naturalistic Scenes. *Frontiers in Psychology*, *08*. https://doi.org/10.3389/fpsyg.2017.00226

Gifford, A. T., Dwivedi, K., Roig, G., & Cichy, R. M. (2022). A large and rich EEG dataset for modeling human visual object recognition. *NeuroImage*, *264*, 119754. https://doi.org/10.1016/j.neuroimage.2022.119754

Gilbert, F., Yang, F., Smith, E., Gardy, S., Farkas, A., Ding, M., Fang, R., & Keil, A. (2025). *Emotional Responses to Naturalistic and AI-generated Affective Pictures: A Systematic Comparison*. Neuroscience. https://doi.org/10.1101/2025.11.03.686276

Grootswagers, T., Zhou, I., Robinson, A. K., Hebart, M. N., & Carlson, T. A. (2022). Human EEG recordings for 1,854 concepts presented in rapid serial visual presentation streams. *Scientific Data*, *9*(1), 3. https://doi.org/10.1038/s41597-021-01102-7

Hebart, M. N., Contier, O., Teichmann, L., Rockter, A. H., Zheng, C. Y., Kidder, A., Corriveau, A., Vaziri-Pashkam, M., & Baker, C. I. (2023). THINGS-data, a multimodal collection of large-scale datasets for investigating object representations in human brain and behavior. *eLife*, *12*, e82580. https://doi.org/10.7554/eLife.82580

Hebart, M. N., Dickter, A. H., Kidder, A., Kwok, W. Y., Corriveau, A., Van Wicklin, C., & Baker, C. I. (2019). THINGS: A database of 1,854 object concepts and more than 26,000 naturalistic object images. *PLOS ONE*, *14*(10), e0223792. https://doi.org/10.1371/journal.pone.0223792

Kappenman, E. S., Farrens, J. L., Zhang, W., Stewart, A. X., & Luck, S. J. (2021). ERP CORE: An open resource for human event-related potential research. *NeuroImage*, *225*, 117465. https://doi.org/10.1016/j.neuroimage.2020.117465

Mendonça, F., Mostafa, S. S., Morgado-Dias, F., & Ravelo-García, A. G. (2023). On the Use of Kullback–Leibler Divergence for Kernel Selection and Interpretation in Variational Autoencoders for Feature Creation. *Information*, *14*(10), 571. https://doi.org/10.3390/info14100571

Neymotin, S. A., Daniels, D. S., Caldwell, B., McDougal, R. A., Carnevale, N. T., Jas, M., Moore, C. I., Hines, M. L., Hämäläinen, M., & Jones, S. R. (2020). Human Neocortical Neurosolver (HNN), a new software tool for interpreting the cellular and network origin of human MEG/EEG data. *eLife*, *9*, e51214. https://doi.org/10.7554/eLife.51214

Pastor, M. C., Bradley, M. M., Löw, A., Versace, F., Moltó, J., & Lang, P. J. (2008). Affective picture perception: Emotion, context, and the late positive potential. *Brain Research*, *1189*, 145-151. https://doi.org/10.1016/j.brainres.2007.10.072

Schindler, S., Bruchmann, M., & Straube, T. (2023). Beyond facial expressions: A systematic review on effects of emotional relevance of faces on the N170. *Neuroscience & Biobehavioral Reviews*, *153*, 105399. https://doi.org/10.1016/j.neubiorev.2023.105399

Seijdel, N., Loke, J., Van De Klundert, R., Van Der Meer, M., Quispel, E., Van Gaal, S., De Haan, E. H. F., & Scholte, H. S. (2021). On the Necessity of Recurrent Processing during Object Recognition: It Depends on the Need for Scene Segmentation. *The Journal of Neuroscience*, *41*(29), 6281-6289. https://doi.org/10.1523/JNEUROSCI.2851-20.2021

Vidaurre, D. (2024). A generative model of electrophysiological brain responses to stimulation. *eLife*, *12*, RP87729. https://doi.org/10.7554/eLife.87729.3

Yamazaki, K., Vo-Ho, V.-K., Bulsara, D., & Le, N. (2022). Spiking Neural Networks and Their Applications: A Review. *Brain Sciences*, *12*(7), 863. https://doi.org/10.3390/brainsci12070863

UF UNIVERSITY of FLORIDA

College of Liberal Arts and Sciences
Department of Psychology
Laboratory for Brain, Body, Behavior

Gainesville, FL 32611
Phone: 352-273-0843
6/8/2026

REVIEWER COMMENTS

Reviewer #1 (Remarks to the Author):

I am happy that the reviewers have addressed all my comments. I recommend acceptance.

Response: The authors would like to thank the reviewer for their time and effort that went into reviewing this manuscript.

Reviewer #2 (Remarks to the Author):

The authors have done substantial additional work to properly train and test the performance of the model. This is useful for readers to understand its strengths and limitations. I am still concerned that some results are over-stated or not correctly contextualized and that this may misguide readers. Specifically, the new out of distribution tests done on the THINGS and ERP-Core datasets are important tests, but the description by the authors does not acknowledge the substantially lower correlation between the model and data compared to the within distribution test results. Rather, these results are described as "strong". The authors should accurately acknowledge that out of distribution performance is weaker, and that users of Concept2Brain should proceed carefully as a result.

We thank the reviewer for raising this point. We understand the reviewer's concern and agree that the results need toning down. Indeed, the correlation of the out-of-sample examples (THINGS and ERP-Core) is substantially lower compared to the within test correlations derived from the cross-validation. As recommended by the reviewer, we have toned down the interpretations in the Results section. In addition, we have emphasized the need for caution and careful use of the Concept2Brain tool in the Discussion section.

Relatedly, the paper should include a much clearer disclaimer that the usage of text as input has not been verified in anyway, and so this use case is possible with this model, but not validated.

We agree that the use of texts as inputs in the Concept2Brain is less validated. Consequently, we have added a note of caution, as recommended, regarding the usage of texts as input, in the Results and Discussion sections. Specifically, we state that the text information may not predictably correspond with picture information and thus caution must be taken when using this option.

Given that the authors are now correctly cross-validating their model by using held out images, I am slightly confused about which version of the model is used for the web interface. Specifically, for the results in Figures 1 and 2, 10 different versions of the model were trained (one for each fold). Which version of the model is run when using the interface?

Thank you for raising this crucial point. We agree on the confusion regarding which model is used in the web interface and apologize for it. For the cross-validation analysis, we used the same model that was trained using 288 pictures (80%), leaving the rest for evaluation and for hold-out-validation. However, to optimize the performance of the web interface, it uses the original model trained on the 360 pictures. Both models were compared, and the model that was trained on all pictures showed a slightly less noisy output, which also converged strongly with the cross-validation model. It was thus used as basis for the web interface. This information has now been added in the "The Concept2Brain cloud platform" section of the Methods.

The authors would like to thank the reviewer for their time invested in this review and all the useful feedback provided.

Reviewer #3 (Remarks to the Author):

The authors have responded to my questions satisfactorily for the most part.

I'd like to follow up on point number 4, though.

"The newly added analysis of oscillatory content (Figure 3D) demonstrates that some information from the individual trials, such as aspects of induced oscillations, is contained in the synthetic data".

A time-frequency analysis of the data is totally based on averages, so there's no account of trial-specific variability in there - unless I'm missing something?

The reviewer is correct. This question is related to earlier questions regarding the nature of the output of Concept2brain as single-trial EEG segments versus averages. We wished to convey the observation that many "induced" power changes are visible in the Concept2Brain output such as alpha power reduction, which often is not observed when pipelining ERPs into time-frequency analysis. We have completely removed this section and associated speculation of single-trial variability to avoid confusion.

"In the revised manuscript, we acknowledge this fact and emphasize that the lack of single-trial variability in response to the same stimulus represents a problem for many advanced single-trial analyses".

Apologies if I have missed something but I can't find any of this in the new text. Is it possible that it slipped?

Thanks for catching this, we have paraphrased this in a confusing fashion (see comment above) in the prior version and have now stated the nature of the simulated signal aspect more clearly, at the beginning and note specific limitations throughout the text.

Once again, thanks for your patience and constructive comments throughout this review process.
