# Supplementary material for: Concept2Brain: an AI model for predicting neurophysiological responses to text and pictures
Source: Nat Commun. 2026 Jul 23;17:8961. doi: 10.1038/s41467-026-75653-x (PMC13503920; doi:10.1038/s41467-026-75653-x)
Supplement: Supplementary file 3 [file 41467_2026_75653_MOESM3_ESM.pdf]

nature portfolio

Corresponding author(s): Alejandro Santos-Mayo

Last updated by author(s): Jun 8, 2026

Reporting Summary

Nature Portfolio wishes to improve the reproducibility of the work that we publish. This form provides structure for consistency and transparency in reporting. For further information on Nature Portfolio policies, see our Editorial Policies and the Editorial Policy Checklist.

Statistics

For all statistical analyses, confirm that the following items are present in the figure legend, table legend, main text, or Methods section.

n/a	Confirmed	
☐	☒	The exact sample size (n) for each experimental group/condition, given as a discrete number and unit of measurement
☐	☒	A statement on whether measurements were taken from distinct samples or whether the same sample was measured repeatedly
☐	☒	The statistical test(s) used AND whether they are one- or two-sided<br>*Only common tests should be described solely by name; describe more complex techniques in the Methods section.*
☒	☐	A description of all covariates tested
☒	☐	A description of any assumptions or corrections, such as tests of normality and adjustment for multiple comparisons
☐	☒	A full description of the statistical parameters including central tendency (e.g. means) or other basic estimates (e.g. regression coefficient) AND variation (e.g. standard deviation) or associated estimates of uncertainty (e.g. confidence intervals)
☐	☒	For null hypothesis testing, the test statistic (e.g. F, t, r) with confidence intervals, effect sizes, degrees of freedom and P value noted<br>*Give P values as exact values whenever suitable.*
☒	☐	For Bayesian analysis, information on the choice of priors and Markov chain Monte Carlo settings
☒	☐	For hierarchical and complex designs, identification of the appropriate level for tests and full reporting of outcomes
☐	☒	Estimates of effect sizes (e.g. Cohen's d, Pearson's r), indicating how they were calculated

Our web collection on statistics for biologists contains articles on many of the points above.

Software and code

Policy information about availability of computer code

Data collection	Data was collected using a 128-channel HydroCell net and an EGI Net Amps 300 amplifier. Code used for data preprocessing can be found at: https://github.com/csea-lab/csea-lab
Data analysis	The AI architecture was designed using Pytorch 2.4.0. Python scripts for modeling, training, and evaluation, along with MATLAB code for visualization are available at https://github.com/ASantosMayo/Concept2Brain. The trained models and EEG dataset are accessible at https://osf.io/7dpgm. The architecture leverages OpenAI's contrastive language-image pre-training (CLIP) model, publicly available at: https://github.com/openai/CLIP. The cloud platform was developed with React and a Django rest framework backend. Pytorch and the MNE library were also used for the platform, accesible at: https://concept2brain.brainbodybehavior.science.

For manuscripts utilizing custom algorithms or software that are central to the research but not yet described in published literature, software must be made available to editors and reviewers. We strongly encourage code deposition in a community repository (e.g. GitHub). See the Nature Portfolio guidelines for submitting code & software for further information.

Data

Policy information about availability of data

All manuscripts must include a data availability statement. This statement should provide the following information, where applicable:

- Accession codes, unique identifiers, or web links for publicly available datasets
- A description of any restrictions on data availability
- For clinical datasets or third party data, please ensure that the statement adheres to our policy

Data is open-access available at the following repository: https://osf.io/7dpgm

Research involving human participants, their data, or biological material

Policy information about studies with human participants or human data. See also policy information about sex, gender (identity/presentation), and sexual orientation and race, ethnicity and racism.

Reporting on sex and gender	The present work focus on the new AI architecture, and sex or gender differences were not taking into consideration. Data used for training the model contained human brain electrophysiological recordings from both male and female subjects. In the manuscript, biological sex of the participants is merely informed, yet not used for any further analysis.
Reporting on race, ethnicity, or other socially relevant groupings	No variables such as race, ethnicity nor social status were used in the manuscript.
Population characteristics	n/a
Recruitment	Participants collected in the original EEG studies were recruited from university class groups. Course credits were given in compensation for their participation.
Ethics oversight	Procedures of both datasets were approved by the University of Florida institutional review board and informed conset was signed by all participants in accordance with the Declaration of Helsinki.

Note that full information on the approval of the study protocol must also be provided in the manuscript.

Field-specific reporting

Please select the one below that is the best fit for your research. If you are not sure, read the appropriate sections before making your selection.

☒ Life sciences ☐ Behavioural & social sciences ☐ Ecological, evolutionary & environmental sciences

For a reference copy of the document with all sections, see nature.com/documents/nr-reporting-summary-flat.pdf

Life sciences study design

All studies must disclose on these points even when the disclosure is negative.

Sample size	Two datasets of EEG recordings were employed, involving 45 and 43 subjects.
Data exclusions	Only those noisy trials showing as outliers were automatically discarded using the statistical Control of Artifacts in Dense Array EEG/MEG Studies (SCDADS).<br>No other exclusion criteria were followed in the data collection.
Replication	The AI model performance was checked by using similar pictures to those use in the training phase. Such out-of-sample comparison shed a Pearson's correlation of r = .23 (p < 0.0001).
Randomization	No experimental groups were used. All EEG recordings form all participants were used as training dataset for the AI model.
Blinding	No experimental groups were used.

Reporting for specific materials, systems and methods

We require information from authors about some types of materials, experimental systems and methods used in many studies. Here, indicate whether each material, system or method listed is relevant to your study. If you are not sure if a list item applies to your research, read the appropriate section before selecting a response.

Materials & experimental systems

n/a	Involved in the study
☒	☐ Antibodies
☒	☐ Eukaryotic cell lines
☒	☐ Palaeontology and archaeology
☒	☐ Animals and other organisms
☒	☐ Clinical data
☒	☐ Dual use research of concern
☒	☐ Plants

Methods

n/a	Involved in the study
☒	☐ ChIP-seq
☒	☐ Flow cytometry
☒	☐ MRI-based neuroimaging

Plants

Seed stocks	n/a
Novel plant genotypes	n/a
Authentication	n/a
